# Cytotoxic Polyketides from a Deep-Sea Sediment Derived Fungus *Diaporthe phaseolorum* FS431

**DOI:** 10.3390/molecules24173062

**Published:** 2019-08-22

**Authors:** Zheng Niu, Yuchan Chen, Heng Guo, Sai-Ni Li, Hao-Hua Li, Hong-Xin Liu, Zhaoming Liu, Weimin Zhang

**Affiliations:** State Key Laboratory of Applied Microbiology Southern China, Guangdong Provincial Key Laboratory of Microbial Culture Collection and Application, Guangdong Open Laboratory of Applied Microbiology, Guangdong Institute of Microbiology, Guangdong Academy of Sciences, Yuexiu District, Guangzhou 510070, China

**Keywords:** *Diaporthe phaseolorum*, deep-sea derived fungus, polyketides, cytotoxicity

## Abstract

Two new chromone-derived polyketides phaseolorins, G and H (**1** and **2**), and one new anthraquinone derivative, phaseolorin I (**3**), together with three known compounds (**4**–**6**), were isolated from the deep-sea sediment-derived fungus *Diaporthe phaseolorum* FS431. The structures of the new compounds were determined by comprehensive analysis of their spectroscopic data, and the absolute configuration of **1** was established by quantum chemical calculations of electron capture detection (ECD). All the isolated compounds (**1**–**6**) were tested for their in vitro cytotoxic activities against four human tumor cell lines, of which compound **4** exhibited significant effect against MCF-7, HepG-2, and A549 tumor cell lines with IC_50_ values of 2.60, 2.55, and 4.64 µM, respectively.

## 1. Introduction

Secondary metabolites from marine fungi have attracted the attention of chemists and biologists because of their unique chemical structures and significant biological activities [1]. In past decades, large numbers of novel chemical structures have been found from deep-sea fungi, such as polyketides, terpenoids, steroids, peptides, alkaloids, and so on [2,3]. Furthermore, polyketides have not only dominant numbers in structures but also diverse potent bioactivities, including cytotoxic, antibacterial, antifungal, radical scavenging, and antiviral [4,5,6].

The genus *Diaporthe* is one of the most common and widely distributed fungal taxa, which predominantly produces a large number of polyketides. Because of its importance as plant pathogen, the genus has been thoroughly investigated. Its secondary metabolites were collected from a wide range of species derived from different habitats, which were mostly obtained from endophytic fungi. Their metabolites are full of diverse natural products, such as diketopiperazines [7], triterpenoid [8], bisanthraquinones [9], highly substituted benzophenones [10], lactons [11], xanthones [12], and mycotoxins [12], which exhibit anti-IAV [6], anti-angiogenic [7], antimalarial [12], antihyperlipidemic [13] activities, and so on.

**Figure 1:**
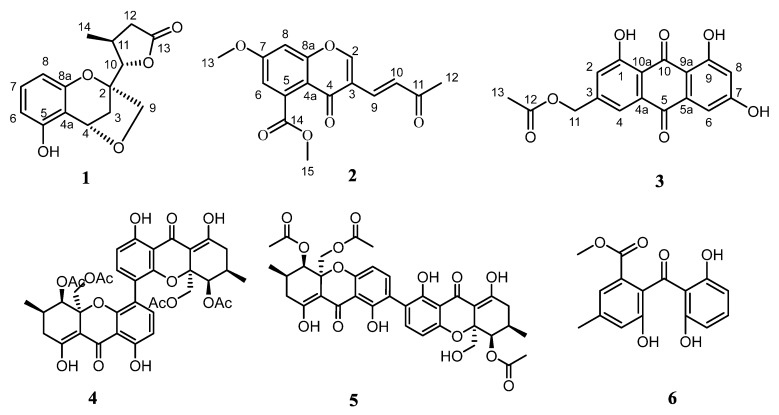
Chemical structures of compounds **1**–**6**.

However, the investigation of the genus *Diaporthe* from deep-sea was rarely reported. In our ongoing effort to search for bioactive metabolites from the deep-sea derived fungi, a strain of *D. phaseolorum* FS431 was collected from a sediment sample of the Indian Ocean, and its chemical investigation led to the isolation and the identification of three new polyketides, named phaseolorin G-I (**1**–**3**), along with three known compounds, phomoxanthone A (**4**) [14], dicerandrol B (**5**) [15], and 2,2′,6′-trihydroxy-4-methyl-6-methoxy-acyl-diphenylmethanone (**6**) (Figure 1) [16]. All the isolated compounds were tested for their in vitro cytotoxic activities against four human tumor cell lines. Herein, the details of isolation, structure identification, as well as cytotoxicity assay are reported.

## 2. Results and Discussion

### 2.1. Structure Elucidation

Compound **1** was isolated as yellow oil. Its molecular formula was established to be C_15_H_16_O_5_ on the basis of high-resolution electrospray ionization mass spectroscopy (HRESIMS) at *m/z* 275.0926 [M − H]^−^ (calcd for 275.0919), indicating eight degrees of unsaturation. The IR absorption at 3225 and 1740 cm^−1^ suggested the presence of hydroxy and carbonyl groups, respectively. The ^1^H-NMR spectrum of **1** revealed the presence of a phenolic hydroxyl signal at δ_H_ 8.59 (1H, br s, 1-OH), three ABX coupling aromatic protons at [δ_H_ 6.42 (1H, br d, *J* = 8.1 Hz, H-6), 6.97 (1H, t, *J* = 8.1 Hz, H-7), 6.31 (1H, br d, *J* = 8.1 Hz, H-8)], three methylenes at [δ_H_ 4.05 (2H, s, H-9), 2.22 (1H, m, H-3a), 2.12 (1H, m, H-3b), 2.85 (1H, overlapped, H-12a), 2.24 (1H, m, H-12b)], three methines at [δ_H_ 5.44 (1H, d, *J* = 5.0 Hz, H-4), 4.51 (1H, d, *J* = 4.0 Hz, H-10), 2.08 (1H, m, H-11)], and a methyl group at δ_H_ 1.28 (3H, d, *J* = 6.1 Hz, H_3_-14). The ^13^C-NMR combined with HSQC spectra (Table 1) of **1** resolved 15 carbon signals composed of a carbonyl carbon (δ_C_ 176.4), one methyl, three methylenes, six aromatic carbonyls, three sp^3^ methines, and three aliphatic quaternary carbons. Comprehensive analysis of the 1D-NMR data indicated the existence of *thio*-trisubstituted benzene ring in **1**, which was similar to those constructed the same backbone [17].

**Figure 2:**
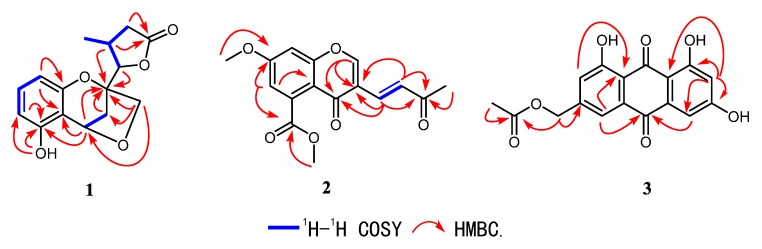
Key ^1^H–^1^H COSY and HMBC correlations for compounds **1**–**3**.

The ^1^H–^1^H COSY spectrum (Figure 2) indicated the presence of four coupling fragments H-6/H-7/H-8, H-3/H-4, H-10/H-11/H-12 and H-11/H-14. The HMBC correlations (Figure 2) from H-8 to C-4a/C-8a and from H-6 to C-5/C-4a further evidenced the *thio*-trisubstituted benzene ring. Meanwhile, the correlations from H-4 to C-4a/C-5/C-8a, H-2 to C-8a, and 5-OH to C-5 suggested a benzopyran skeleton with a phenol group substituted at C-5. Moreover, the HMBC cross peaks from H_3_-14 to C-10/C-12, H-11/H-12 to C-13, and H-10 to C-13 combined with the downfield shifted of H-10 and C-10 (δ_H_ 4.51; δ_C_ 88.7) further confirmed a γ-lactone ring, which was connected to C-2 on the basis of the correlation from H-10/H-11 to C-2. Finally, the correlation between H_2_-9 and H-4 suggested the etherification from the hydroxymethyl to C-4, which was also supported by the degrees of unsaturation. Hence, the planar structure of **1** was established as shown in Figure 1.

**Figure 3:**
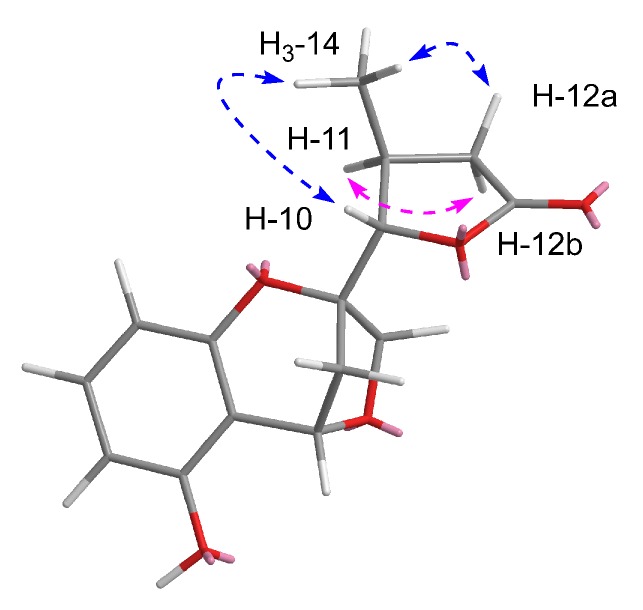
Key rotating overhauser effect (ROE) correlations of compound **1**.

The relative configuration between C-10 and C-11 position was deduced by the rotating overhauser effect spectroscopy (ROESY) data. The cross-peaks (Figure 3) of H-10/H_3_-14/H-12a and H-11/H-12b suggested the *trans* configuration of H-10 and H-11. However, due to the lack of ROESY effects between H-4and H_2_-9, the relative configurations of C-2 and C-4 could not be identified unambiguously.

In order to establish the complete relative configuration, the quantum chemical calculations of the NMR data of the possible diastereomers, **1a** and **1b**, were performed. Initially, an exhaustive conformation searching was conducted by Merck Molecular Force Field (MMFF) in Spartan′16 (Wavenfunction, Irvine, CA, USA). Conformers with energy less than 10 kcal mol^−1^ energy window were generated and reoptimized using DFT (Density Functional Theory) calculations at the b3lyp/6-31+g (d, p) level. Frequency calculations were performed at the same level to confirm that each optimized conformer was true minimum and to estimate their relative thermal free energy (ΔG) at 298.15 K.

**Figure 4:**
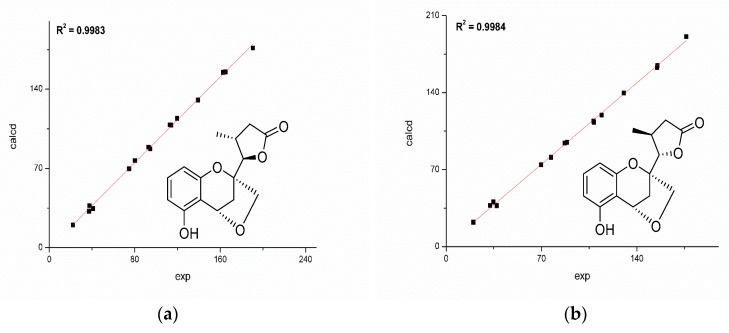
Correlations between experimental and calculated ^13^C-NMR chemical shifts of **1a** (**a**) and **1b** (**b**).

The reoptimized conformers were further subjected to ^13^C-NMR chemical shifts at the mPW1PW91-SCRF/6-311+G (d, p) level in acetone. The result indicated that both **1a** and **1b** shared similar correlation coefficients (R^2^: **1a** 0.9983; **1b** 0.9984, Figure 4), but the DP4^+^ (carbon data) simulation suggested **1b** should be the true candidate with 74.12% probability compared with those of **1a** (22.88% probability). Besides, **1a** exhibited a mean absolute error (MAE) value of 1.6 ppm, while **1b** showed a slightly better value of 1.5 ppm.

**Figure 5:**
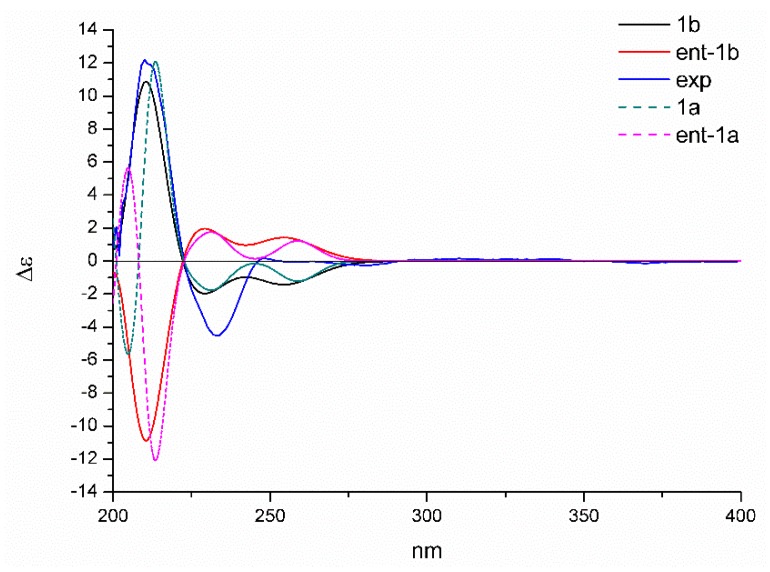
Calculated and experimental electron capture detection (ECD) spectra of **1** in methanol.

Moreover, the absolute configuration could be established by the electron capture detection (ECD) calculation. The reoptimized conformers in the previous NMR calculations were selected, and the theoretical spectra of **1a**/ent-**1a** and **1b**/ent-**1b** were generated at b3lyp/6-311+g (d, p) level. The result showed that the calculated plot of 2*S*,4*S*,10*S*,11*S*-**1** (**1b**) gave the best agreement with the experimental spectrum (Figure 5), which exhibited the key positive Cotton effect at 210 nm and the negative Cotton effect at 230 nm. Hence, the absolute configuration of **1** was determined to be 2*S*,4*S*,10*S*,11*S*-**1**.

Compound **2** was obtained as white crystals. Its molecular formula was established to be C_16_H_14_O_6_ on the basis of HRESIMS at *m/z* 303.0866 [M + H]^+^ (calcd for 303.0869), indicating ten degrees of unsaturation. The UV absorption at 279 nm and IR peak at 1636 cm^−1^ suggested the existence of α, β-unsaturated carbonyl group. The ^1^H-NMR spectrum of **2** revealed a methine group at δ_H_ 8.08 (1H, s, H-2), a double bond at [δ_H_ 7.41 (1H, d, *J* = 16.1 Hz, H-11), 7.29 (1H, d, *J* = 16.1 Hz, H-10)], two meta-coupled aromatic protons at [δ_H_ 6.96 (1H, d, *J* = 2.4 Hz, H-6), 6.92 (1H, d, *J* = 2.4 Hz, H-8)], two methoxyl signals at at [δ_H_ 4.03 (3H, s, H_3_-15), 3.93 (3H, s, H_3_-13)], and a methyl signal at 2.35 (3H, s, H_3_-12). The ^13^C-NMR (Table 2) combined with HMBC spectra (Figure 2) of **2** revealed 16 carbon signals, including one methyl, two methoxyls, five sp^2^ methines, and eight quaternary carbons including three carbonyl carbons at δ_C_ at 198.7, 174.2, and 169.2. By comparing the 1D-NMR data with those of diaporchromanones A–D, which were obtained as isomers from *Diaporthe phaseolorum*, compound **2** should have constructed a chromanone backbone but with a dehydrated side chain [18]. The ^1^H–^1^H COSY spectrum showed the presence of one coupling fragment, H-10/H-11. The HMBC correlations (Figure 2) from H-2 to C-3/C-4/C-8a, H-8 to C-7/C-8a, and H-6 to C-4a established the core structure of a chroman-4-one. Moreover, the HMBC cross peaks from H-6/H-15 to C-14 and H-13 to C-7 indicated a chroman-4-one skeleton, which was substituted with an ester group at C-5 and a methoxyl group at C-7. From H-9 to C-3/C-4, H-10 to C-3, a double bond was linked with C-3. Meanwhile, the geometry of the Δ_9,10_ was deduced to be *E* based on the large coupling constants (*J* = 16.1 Hz). Finally, according to the correlations from H-9/H-10/H_3_-12 to C-11, an acetyl was elucidated. The above mentioned data led to the establishment of the structure of **2**.

Compound **3** was isolated as a yellow oil. Its molecular formula was established to be C_17_H_12_O_7_ on the basis of HRESIMS at *m/z* 327.0514 [M − H]^−^ (calcd for 327.0504), indicating twelve degrees of unsaturation. UV spectrum exhibited an obvious absorption band at 434 nm, indicating a large conjugated system in **3**. The ^1^H-NMR spectrum (Table 3) of **3** revealed two phenol signals at [δ_H_ 12.12 (1H, br s, 1-OH), 12.12 (1H, br s, 9-OH)], an aromatic ring system present at [δ_H_ 7.63 (1H, br s, H-2), 7.31 (1H, br s, H-4)], and [δ_H_ 7.12 (1H, d, *J* = 2.4 Hz, H-6), 6.58 (1H, d, *J* = 2.4 Hz, H-8)], and one methyl signal at δ_H_ 2.14 (3H, s, H-13). The ^13^C-NMR (Table 3) and the HMBC spectra (Figure 2) of **3** revealed 17 carbon signals, one methyl, one sp^3^ methylenes, four sp^2^ methines, and eleven quaternary carbons including three carbonyl carbons at δ_C_ 189.4, 181.3, and 170.2. The key HMBC ^3^J correlations from H-4, H-6 to C-5, H-2/H-4 to C-10a, H-8 to C-6/C-9a along with weak ^4^J signal from H-4 to C-10 helped define the central anthracene-9,10-dione ring. The relations from H-11 to C-3/C-12 and H_3_-13 to C-12 established the fragment of methyl acetate at C-3. Moreover, the characteristic downfield chemical shifts of 1-OH and 9-OH (δ_H_ 12.12 ppm) suggested the substituted location at C-1 and C-9, forming hydrogen bonds with the carbonyl carbon (C-10).

### 2.2. Cytotoxicity Activity

Compounds **1**–**6** were evaluated for their cytotoxicities against four human cancer cell lines: SF-268, MCF-7, HepG-2, and A549. Compound **4** was found to exhibit significant effects against MCF-7, HepG-2, and A549, with IC_50_ values of 2.60, 2.55, and 4.64 μM, respectively (Table 4).

## 3. Materials and Methods

### 3.1. General Experimental Procedures

UV spectra were recorded on a Shimadzu UV-2600 spectrophotometer (Shimadzu, Kyoto, Japan). IR data were acquired on a Shimadzu IR Affinity-1 spectrometer (Shimadzu, Kyoto, Japan). 1D- and 2D-NMR spectra were obtained on a Bruker AVANCE IIITM HD 600 MHz NMR spectrometer (Bruker, Fällanden, Switzerland) using TMS as an internal standard. HRESIMS and electrospray ionization-mass spectrometry (ESIMS) data were measured, respectively, on a Thermo MAT95XP high resolution mass spectrometer (Thermo Fisher Scientific, Bremen, Germany) and an Agilent Technologies 1290-6430A Triple Quad LC/MS (Agilent Technologies, Palo Alto, CA, USA). Optical rotations were obtained on an Anton Paar MCP-500 spectropolarimeter (Anton Paar, Graz, Austria) with MeOH as solvent at 25 °C. CD spectra were determined using a Jasco 820 spectropolarimeter (Jasco Corporation, Kyoto, Japan). Preparative HPLC collection used a C_18_ column (YMC-pack ODS-A, 250 × 20 mm, 5 µm, 12 nm, YMC Co., Ltd., Kyoto, Japan). Semipreparative HPLC separations were performed utilizing a C_18_ column (YMC-pack ODS-A/AQ, 250 × 10 mm, 5 µm, 12 nm, YMC CO., Ltd., Kyoto, Japan). Column chromatography (CC) was performed on silica gel (200–300 mesh, Qingdao Marine Chemical Inc., Qingdao, China) and Sephadex LH-20 (Amersham Biosciences, Uppsala, Sweden). Solvents for isolation were analytical grade (Guangzhou Chemical Regents Company, Ltd., Guangzhou, China). TLC spots were visualized under UV light and by dipping into 10% H_2_SO_4_ in alcohol followed by heating.

### 3.2. Fungal Material

The fungal strain FS431 was isolated from a marine sediment sample collected from the Indian Ocean (depth 3605 m, 7°57.75944′ N, 89°19.43851′ E) in March 2016 and identified as *Diaporthe phaseolorum* based on sequencing of the internal transcribed spacer (ITS) region (Accession No.MK459544) with 99% similarity to *Diaporthe phaseolorum* MJ14 (Accession No.KM203581). The strain was deposited at the Guangdong Provincial Key Laboratory of Microbial Culture Collection and Application, Guangdong Institute of Microbiology.

### 3.3. Fermentation and Extraction

*D. phaseolorum* FS431 was cultured for 5 days at 28 °C in a potato dextrose agar (PDA) culture plate. The mycelial plugs were transferred to ten 500 mL Erlenmeyer flasks, each containing 250 mL potato dextrose broth (20% potato, 2% glucose, 0.3% KH_2_PO_4_, 0.15% MgSO_4_·7H_2_O, and 250 mL water with 1.5% sea salt), and then incubated on a rotary shaker at 120 r/m and 28 °C for 4 days as seed cultures. After that, each of the seed cultures (20 mL) was transferred into autoclaved 3 L Erlenmeyer flasks (total of 10 bottles), each containing 480 g of rice and 600 mL of 3% saline water. All flasks were incubated statically at room temperature for 30 days.

### 3.4. Isolation and Purification

The fermented rice substrate was extracted three times with ethyl acetate (EtOAc), and the combined EtOAc layers were evaporated to dryness under vacuum to afford the EtOAc extract (122.7 g). The EtOAc extract was chromatographed on a silica gel (200–300 mesh) column eluted with a step gradient of petroleum ether (PE)–EtOAc (*v/v* 30:1, 20:1, 10:1, 5:1, 2:1, 1:1, 0:1) and CH_2_Cl_2_–MeOH (*v/v* 20:1, 10:1, 5:1) to give 10 fractions (Fr. 1–Fr. 10). Fr. 6 was chromatographed by ODS column chromatography using gradient elution of H_2_O–MeOH (30–100%) to give 7 subfractions (Fr. 6.1–Fr. 6.7). Fr. 6.3 was purified by silica gel flash column chromatography (PE/EtOAc *v/v* 10:1–2:1) to yield 4 fractions (Fr. 6.3.1–Fr. 6.3.4). Fr. 6.3.4 was subjected to CC on Sephadex LH-20 (CH_2_Cl_2_/MeOH *v*/*v*, 1:1,) and semiprep-HPLC (MeOH/H_2_O *v/v*, 50:50, 3 mL/min) to give compound **1** (3.3 mg, *t*_R_ = 9.2 min) and **6** (10.5 mg, *t*_R_ = 11.8 min). Fr. 6.6 was purified by silica gel flash column chromatography (PE/CH_2_Cl_2_ *v/v* 5:1–1:1) to yield 4 fractions (Fr. 6.6.1–Fr. 6.6.4). Fr. 6.6.2 was purified by Sephadex LH-20 (CH_2_Cl_2_/MeOH, 1:1, *v/v*) to yield compound **3** (5.3 mg). Fr. 6.6.4 was purified by Sephadex LH-20 (CH_2_Cl_2_/MeOH, 1:1, *v/v*) to yield 2 fractions (Fr. 6.6.4.1–Fr. 6.6.4.2), and Fr. 6.6.4.1 was further purified by semiprep-HPLC (MeOH/H_2_O *v/v* 60:40, 3 mL/min) to yield compound **2** (3.2 mg, *t*_R_ = 20.5 min). Fr. 6.6.4.2 was purified by semiprep-HPLC (MeOH/H_2_O, 70:30, *v/v*, 3 mL/min) to yield compound **7** (3.2 mg, *t*_R_ = 13 min). Fr. 6.7 was purified by silica gel flash column chromatography (PE/CH_2_Cl_2_ *v/v* 20:1–5:1) to yield (Fr. 6.7.1–Fr. 6.7.2). Fr. 6.7.2 was then purified by semiprep-HPLC (MeOH/H_2_O *v/v* 90:10, 3 mL/min) to yield compound **4** (23.0 mg, *t*_R_ = 6.3 min) and compound **5** (7.4 mg, *t*_R_ = 7.3 min).

### 3.5. Quantum Chemical Calculation of NMR Chemical Shifts and ECD Spectra

MMFF and DFT/TD-DFT calculations were carried out using the Spartan′14 software (Wavefunction Inc., Irvine, CA, USA) and the Gaussian 09 program, respectively. Conformers that had an energy window lower the 5 kcal·mol^−1^ were generated and optimized using DFT calculations at the b3lyp/6-31+g (d, p) level. Frequency calculations were performed at the same level to confirm that each optimized conformer was true minimum and to estimate their relative thermal free energy (ΔG) at 298.15 K. Conformers with the Boltzmann distribution over 2% were chosen for ^13^C-NMR and ECD calculations at mPW1PW91/6-311+g (d, p) and b3lyp/6-311+g (d, p) level, respectively. Additionally, solvent effects were considered based on the self-consistent reaction field (SCRF) method with the polarizable continuum model (PCM). Details of the conformers’ information were provided in Supporting Information. The ECD spectrum was generated by the SpecDis program using a Gaussian band shape with 0.22 eV exponential half-width from dipole-length dipolar and rotational strengths. The DP4^+^ probability simulations were conducted using an applet available at http://www-jmg.ch.cam.ac.uk/tools/nmr/DP4/.

### 3.6. Cytotoxic Activity Assay

Cytotoxicity of compounds (**1**–**6**) were assayed against four tumor cell lines, including SF-268 (human glioma cell line), MCF-7 (human breast adenocarcinoma cell line), HepG-2 (human liver cell line), and A549 (human lung cancer cell line). Assays were performed by the SRB method [19] with doxorubicin as the positive control. Cells (180 μL) with a density of 30,000 cells/mL of media were injected into 96-well plates and incubated at 37 °C for 24 h under 5% CO2. Then, tested compounds (20 μL) were added. After incubating for 72 h, cell monolayers were fixed with 50 μL trichloroacetic acid (*w*/*v*: 50%) and stained with 0.4% SRB (dissolved in 1% acetic acid) for 30 min. The monolayers were washed by 1% acetic acid three times to remove the unbound dye. Finally, the mixtures were dissolved in 200 µL Tris base solution (10 mM) and recorded the OD at 570 nm using a microplate reader. All data were obtained in triplicate.

### 3.7. Spectroscopic Data

*Phaseolorin G* (**1**): Yellow powder, [α${]}_{D}^{25}$ = +35.4 (c 0.08, MeOH); UV (MeOH) *λ*_max_ (log ε) 205 (3.49), 278 (2.28) nm; CD ∆ε (0.18 mg/mL, MeOH) *λ*_max_ (∆ε) 210 (+12.2), 233 (-4.5), 248 (+0.2) nm; IR (KBr) *ν*_max_ 3225, 2924, 1740, 1260 cm^−1^; ^1^H- and ^13^C-NMR data, see Table 1 and Supplementary Materials; (−)-HRESIMS *m/z* 275.0926 [M − H]^−^ (calcd for C_15_H_15_O_5_ 275.0919).

*Phaseolorin H* (**2**): White crystals, m.p. 289−291 °C; *λ*_ma__x_ (log ε) 207 (3.62), 279 (3.21) nm; IR (KBr) *ν*_max_ 3225, 2920, 1636, 1260 cm^−1^; ^1^H- and ^13^C-NMR data, see Table 2 and Supplementary Materials; (+)-*m/z* 303.0866 [M + H]^+^ (calcd for C_16_H_15_O_6_ 303.0869).

*Phaseolorin I* (**3**): Yellow oil, *λ*_max_ (log ε) 222 (3.14), 265 (2.82), 286 (2.83), 434 (2.61) nm; IR (KBr) *ν*_max_ 3300, 2922, 1609, 1260 cm^−1^; ^1^H- and ^13^C-NMR data, see Table 3 and Supplementary Materials; (−)-HRESIMS *m/z* 327.0514 [M − H]^−^ (calcd for C_17_H_11_O_7_ 327.0504).

## 4. Conclusions

The chemical investigation of the deep-sea derived fungus *D. phaseolorum* FS431 led to isolation of three new polyketides, named phaseolorin G-I (**1**–**3**), together with three known compounds (**4**–**6**). Their structures were elucidated by the detailed analysis of spectroscopic data and quantum chemical calculations. Compound **4** exhibited significant cytotoxic activities against MCF-7, HepG-2, and A549 cancer cell lines with IC_50_ values of 2.60, 2.55, and 4.64 µM, respectively. This study makes a contribution to the chemical diversities of polyketides from deep-sea derived fungi.

## Figures and Tables

**Table 1:** ^1^H-NMR (600 MHz) and ^13^C-NMR (150 MHz) data for **1** in acetone-*d*_6_.

No.	*δ*_H_ (*J* in Hz)	*δ*_C_
2		87.2, C
3a	2.22 (1H, m)	34.9, CH_2_
3b	2.14 (1H, m)	
4	5.44 (1H, d, 5.0)	69.7, CH
4a		114.2, C
5		154.8, C
6	6.42 (1H, br d, 8.0)	108.5, CH
7	6.97 (1H, t, 8.1)	130.4, CH
8	6.31 (1H, br d, 8.0)	108.2, CH
8a		155.2, C
9	4.05 (2H, s)	77.0, CH_2_
10	4.51 (1H, d, 4.0)	88.7, CH
11	2.08 (1H, m)	32.2, CH
12a	2.24 (1H, m)	37.4, CH_2_
12b	2.85 (1H, overlapped)	
13		176.4, C
14	1.28 (3H, d, 6.1)	20.0, CH_3_
5-OH	8.59 (1H, br s)	

**Table 2:** ^1^H-NMR (600 MHz) and ^13^C-NMR (150 MHz) data for **2** in CDCl_3_.

No.	*δ*_H_ (*J* in Hz)	*δ*_C_
2	8.08 (1H, s)	156.5, CH
3		119.6, C
4		174.2, C
4a		114.8, C
5		135.3, C
6	6.96 (1H, d, 2.4)	113.9, CH
7		163.2, C
8	6.92 (1H, d, 2.4)	101.8, CH
8a		157.3, C
9	7.29 (1H, d, 16.1)	133.0, CH
10	7.41 (1H, d, 16.1)	129.7, CH
11		198.7, C
12	2.35 (3H, s)	28.6, CH_3_
13	3.93 (3H, s)	56.2, CH_3_
14		169.2, C
15	4.03 (3H, s)	53.2, CH_3_

**Table 3:** ^1^H-NMR (600 MHz) and ^13^C-NMR (150 MHz) data for **3** in DMSO-*d*_6._

Position	*δ*_H_ (*J* in Hz)	*δ*_C_
1		161.3, C
2	7.30 (1H, br s)	122.3, CH
3		145.6, C
4	7.63 (1H, br s)	117.8, CH
4a		133.3, C
5		181.3, C
5a		135.1, C
6	7.12 (1H, br d, 2.4)	109.2, CH
7		166.2, C
8	6.58 (1H, br d, 2.4)	108.0, CH
9		164.6, C
9a		108.9, C
10		189.4, C
10a		115.1, C
11	5.18 (2H, s)	64.2, CH_2_
12		170.2, C
13	2.14 (3H, s)	20.6, CH_3_
1-OH	12.12 (1H, br s)	161.3, C
9-OH	12.12 (1H, br s)	

**Table 4:** IC_50_ (μM) of compounds **4**–**5** against four tumor cell lines.

NO.	SF-268	MCF-7	HepG-2	A549
**4**	37.86 ± 1.28	2.60 ± 0.28	2.55 ± 0.06	4.64 ± 0.30
**5**	45.26 ± 3.18	48.08 ± 1.55	49.58 ± 0.45	38.64 ± 1.42
**doxorubicin**	0.57 ± 0.04	0.95 ± 0.06	1.18 ± 0.15	0.70 ± 0.04

## Supplementary Materials

The Supplementary Materials are available online. Figures S1–S24: HRESIMS, CD, UV, IR, 1D- and 2D-NMR spectra of compounds **1**–**3**; Figures S25–S30: 1D-NMR spectra of compounds **4**–**6**.
