# Supplementary material for: Cytotoxic Polyketides from a Deep-Sea Sediment Derived Fungus Diaporthe phaseolorum FS431
Source: Molecules. 2019 Aug 22;24(17):3062. doi: 10.3390/molecules24173062 (PMC6749523; doi:10.3390/molecules24173062)
Supplement: Supplementary file 1 [file molecules-24-03062-s001.pdf]

Article

Cytotoxic Polyketides from a Deep-Sea Sediment Derived Fungus *Diaporthe phaseolorum* FS431

Zheng Niu, Yuchan Chen, Heng Guo, Sai-Ni Li, Hao-Hua Li, Hong-Xin Liu, Zhaoming Liu * and Weimin Zhang *

State Key Laboratory of Applied Microbiology Southern China, Guangdong Provincial Key Laboratory of Microbial Culture Collection and Application, Guangdong Open Laboratory of Applied Microbiology, Guangdong Institute of Microbiology, Guangdong Academy of Sciences, Yuexiu District, Guangzhou 510070, China

* Correspondence: liuzm@gdim.cn (Z.L.); wmzhang@gdim.cn (W.Z.); Tel.: +86-20-8768-8309 (W.Z.)

Abstract: Two new chromone-derived polyketides phaseolorins, G and H (**1** and **2**), and one new anthraquinone derivative, phaseolorin I (**3**), together with three known compounds (**4**–**6**), were isolated from the deep-sea sediment-derived fungus *Diaporthe phaseolorum* FS431. The structures of the new compounds were determined by comprehensive analysis of their spectroscopic data, and the absolute configuration of **1** was established by quantum chemical calculations of electron capture detection (ECD). All the isolated compounds (**1**–**6**) were tested for their in vitro cytotoxic activities against four human tumor cell lines, of which compound **4** exhibited significant effect against MCF-7, HepG-2, and A549 tumor cell lines with IC_{50} values of 2.60, 2.55, and 4.64 μM, respectively.

Keywords: *Diaporthe phaseolorum*; deep-sea derived fungus; polyketides; cytotoxicity

Table of Contents

Figure S1. HRESIMS spectrum of Phaseolorin G (**1**)....3
Figure S2. ^{1}H-NMR spectrum (600 MHz, acetone-d_6) of Phaseolorin G (**1**)....3
Figure S3. ^{1}H-NMR spectrum (600 MHz, DMSO-d_6) of Phaseolorin G (**1**)....4
Figure S4. ^{13}C-NMR spectrum (150 MHz, acetone-d_6) of Phaseolorin G (**1**)....5
Figure S5. ^{1}H-^{1}H COSY spectrum (600 MHz, acetone-d_6) of Phaseolorin G (**1**)....5
Figure S6. HSQC spectrum of Phaseolorin G (**1**)....6
Figure S7. HMBC spectrum of Phaseolorin G (**1**)....6
Figure S8. NOESY spectrum (600 MHz, acetone-d_6) of Phaseolorin G (**1**)....7
Figure S9. CD spectrum of Phaseolorin G (**1**)....7
Figure S10. UV spectrum of Phaseolorin G (**1**)....8
Figure S11. IR spectrum of Phaseolorin G (**1**)....8
Figure S12. HRESIMS spectrum of Phaseolorin G (**1**)....9
Figure S13. ^{1}H-NMR spectrum (600 MHz, $CHCl_3$) of Phaseolorin H (**2**)....9
Figure S14. ^{13}C-NMR spectrum (150 MHz, $CHCl_3$) of Phaseolorin H (**2**)....10
Figure S15. ^{1}H-^{1}H COSY spectrum (600 MHz, $CHCl_3$) of Phaseolorin H (**2**)....10
Figure S16. HSQC spectrum of Phaseolorin H (**2**)....11
Figure S17. HMBC spectrum of Phaseolorin H (**2**)....11
Figure S18. UV spectrum of Phaseolorin H (**2**)....12
Figure S19. IR spectrum of Phaseolorin H (**2**)....12
Figure S20. HRESIMS spectrum of Phaseolorin I (**3**)....13
Figure S21. ^{1}H-NMR spectrum (600 MHz, DMSO-d_6) of Phaseolorin I (**3**)....13
Figure S22. ^{13}C-NMR spectrum (150 MHz, DMSO-d_6) of Phaseolorin I (**3**)....14
Figure S23. HMBC spectrum of Phaseolorin I (**3**)....14
Figure S24. UV spectrum of Phaseolorin I (**3**)....15
Figure S25. IR spectrum of Phaseolorin I (**3**)....15
Figure S26. ^{1}H-NMR spectrum (600 MHz, $CHCl_3$) of Phomoxanthone A (**4**)....16
Figure S27. ^{13}C-NMR spectrum (150 MHz, $CHCl_3$) of Phomoxanthone A (**4**)....16
Figure S28. ^{1}H-NMR spectrum (600 MHz, $CHCl_3$) of Dicerandrol B (**5**)....17
Figure S29. ^{13}C-NMR spectrum (150 MHz, $CHCl_3$) of Dicerandrol B (**5**)....17
Figure S30. 1H-NMR spectrum (600 MHz, CD_3OD) of 2,2′,6′-trihydroxy-4-methyl-6-methoxy-acyl-diphenylmethanone (**6**)....18
Figure S31. ^{13}C-NMR spectrum (150 MHz, CD_3OD) of 2,2′,6′-trihydroxy-4-methyl-6-methoxy-acyl-diphenylmethanone (**6**)....18
Table S1. Energy analysis for the Conformers of **1a** (**1**). 19
Figure S32. B3LYP/6-31G(d,p) optimized low-energy conformers of **1a** (**1**)....19
Table S2. Energy analysis for the Conformers of **1b** (**1**). 20
Figure S33. B3LYP/6-31G(d,p) optimized low-energy conformers of **1b** (**1**)....20

Mass Spectrum SmartFormula Report

Analysis Info

Analysis Name	D:\Data\MS\data\201811\niuzheng_Z-28_neg_21_01_5800.d	Acquisition Date	11/22/2018 11:01:17 AM
Method	LC_Direct Infusion_neg_70-500mz.m	Operator	SCSIO
Sample Name	niuzheng_Z-28_neg	Instrument	maXis 255552.00029
Comment			

Acquisition Parameter

Source Type	ESI	Ion Polarity	Negative	Set Nebulizer	0.4 Bar
Focus	Active	Set Capillary	3800 V	Set Dry Heater	180 °C
Scan Begin	70 m/z	Set End Plate Offset	-500 V	Set Dry Gas	4.0 l/min
Scan End	1500 m/z	Set Charging Voltage	0 V	Set Divert Valve	Waste
		Set Corona	0 nA	Set APCI Heater	0 °C

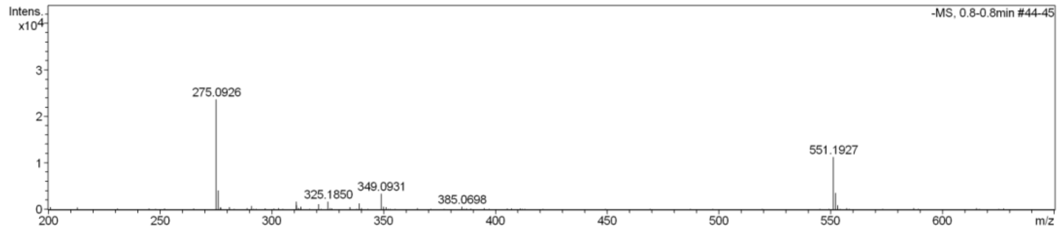

Meas. m/z	#	Ion Formula	Score	m/z	err [ppm]	err [mDa]	mSigma	rdb	e⁻ Conf	N-Rule
275.0926	1	C15H15O5	100.00	275.0925	0.2	0.1	3.8	8.5	even	ok
551.1927	1	C30H31O10	100.00	551.1923	0.7	0.4	8.0	15.5	even	ok

Figure S1. HRESIMS spectrum of Phaseolorin G (**1**)

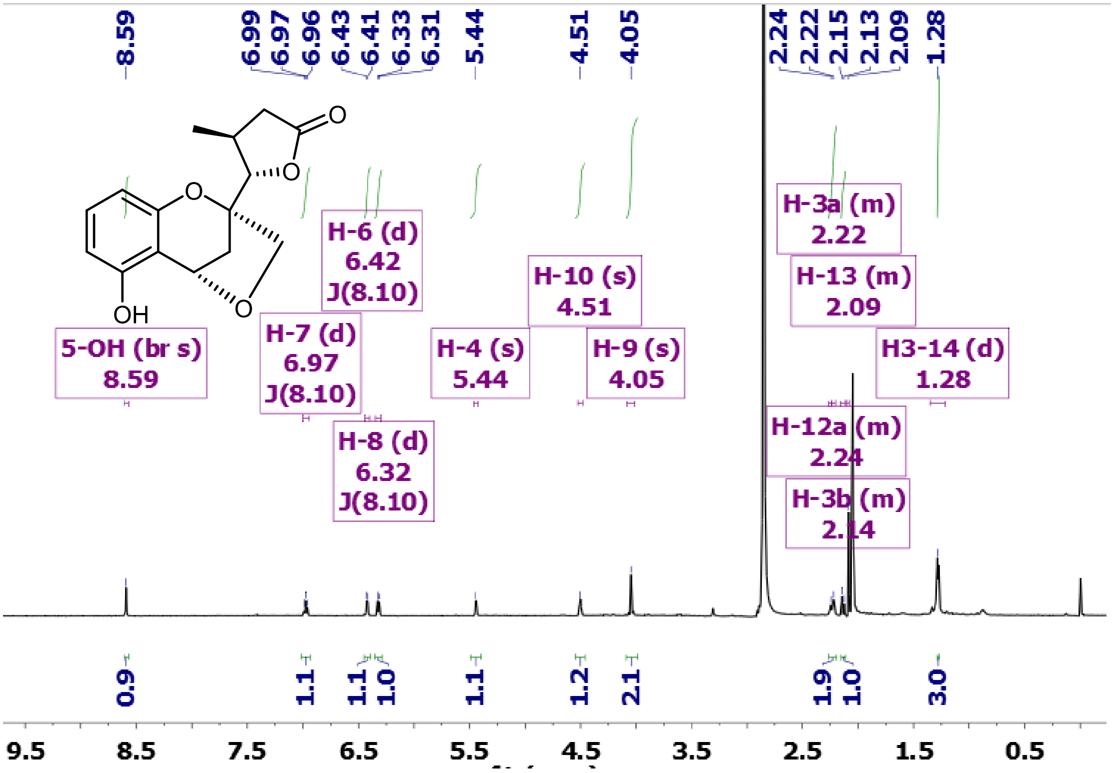

Figure S2. ^{1}H-NMR spectrum (600 MHz, acetone-d_6) of Phaseolorin G (**1**)

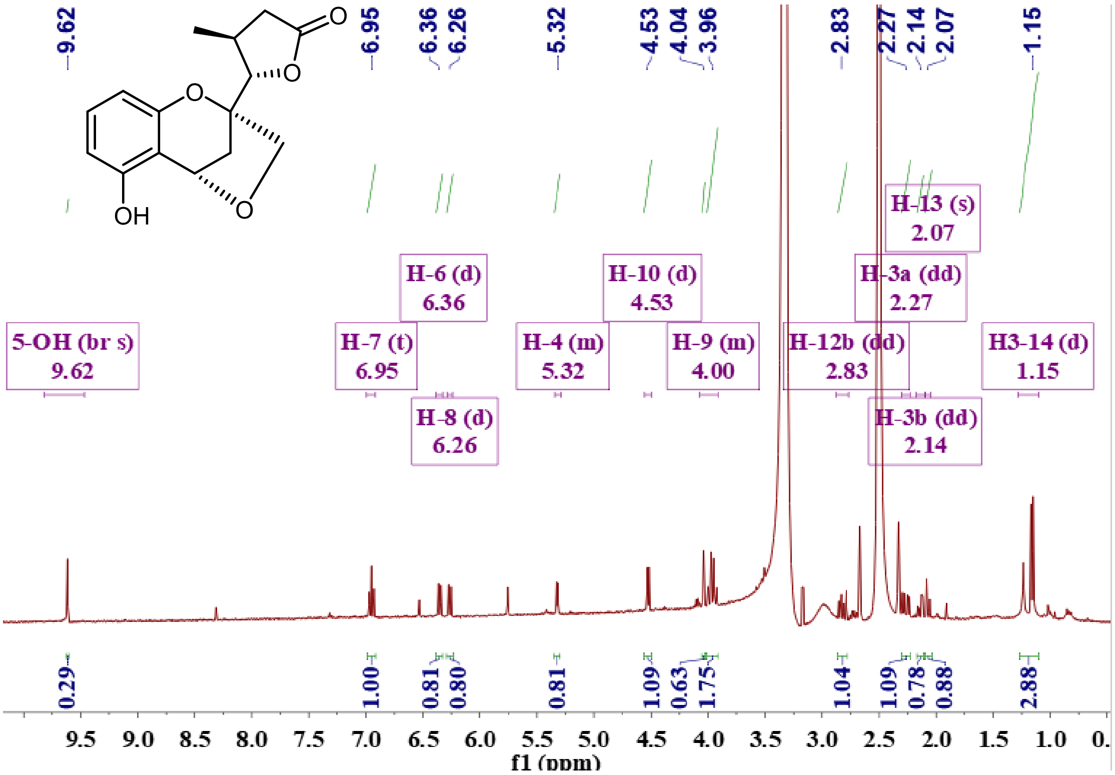

Figure S3. ^{1}H-NMR spectrum (600 MHz, DMSO-d_6) of Phaseolorin G (**1**)

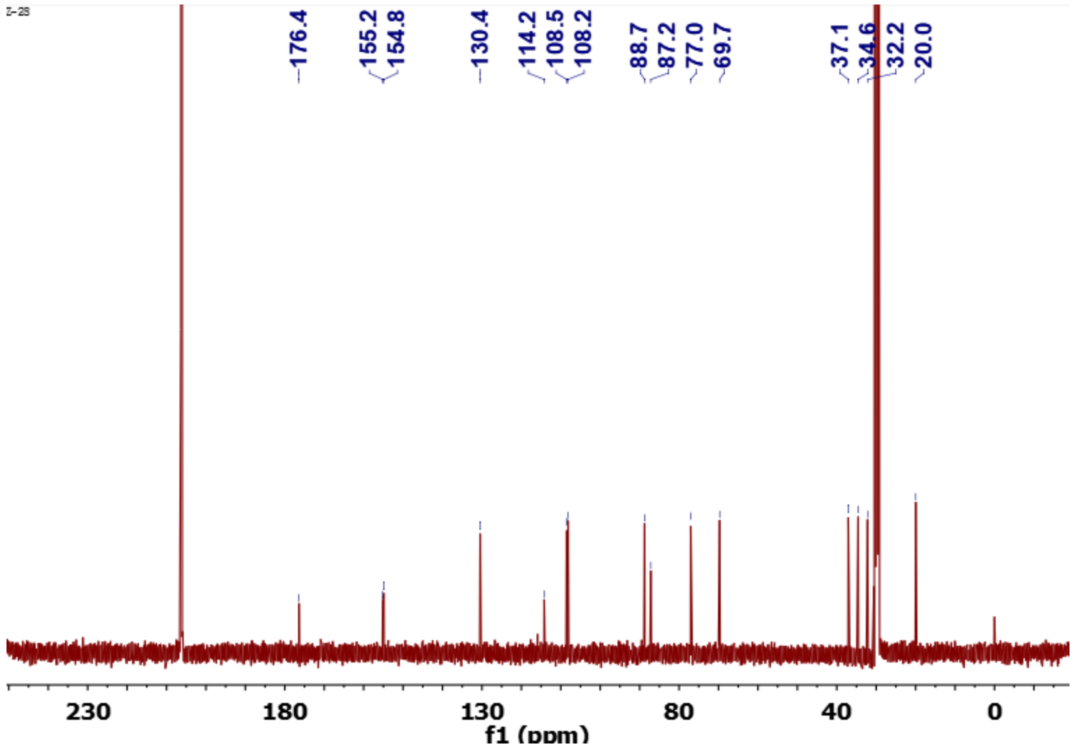

Figure S4. ^{13}C-NMR spectrum (150 MHz, acetone-d_6) of Phaseolorin G (**1**)

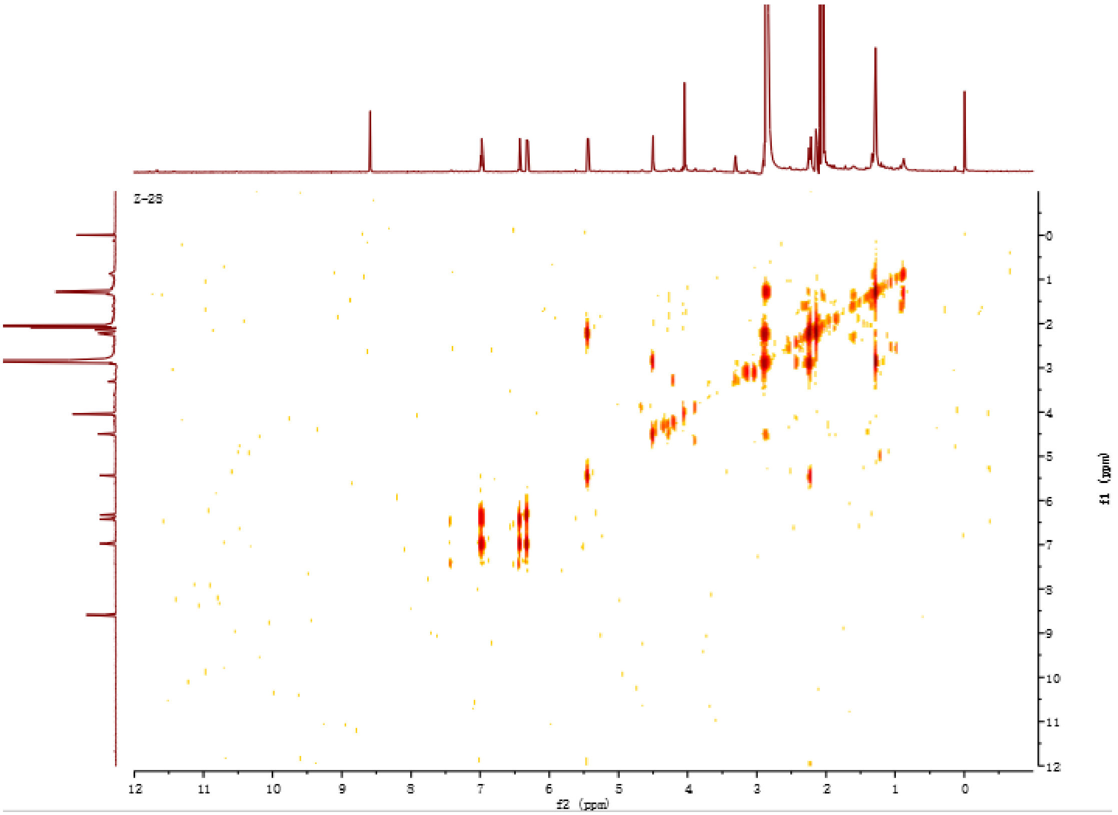

Figure S5. ^{1}H-^{1}H COSY spectrum (600 MHz, acetone-d_6) of Phaseolorin G (**1**)

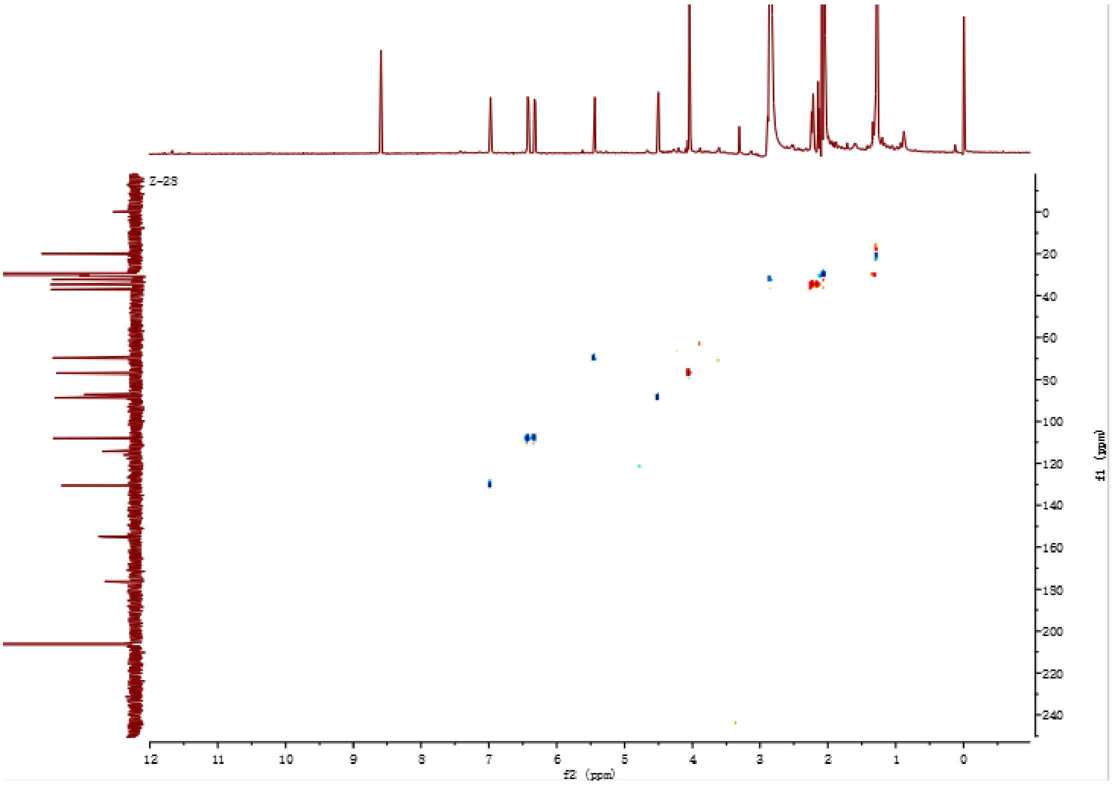

Figure S6. HSQC spectrum of Phaseolorin G (**1**)

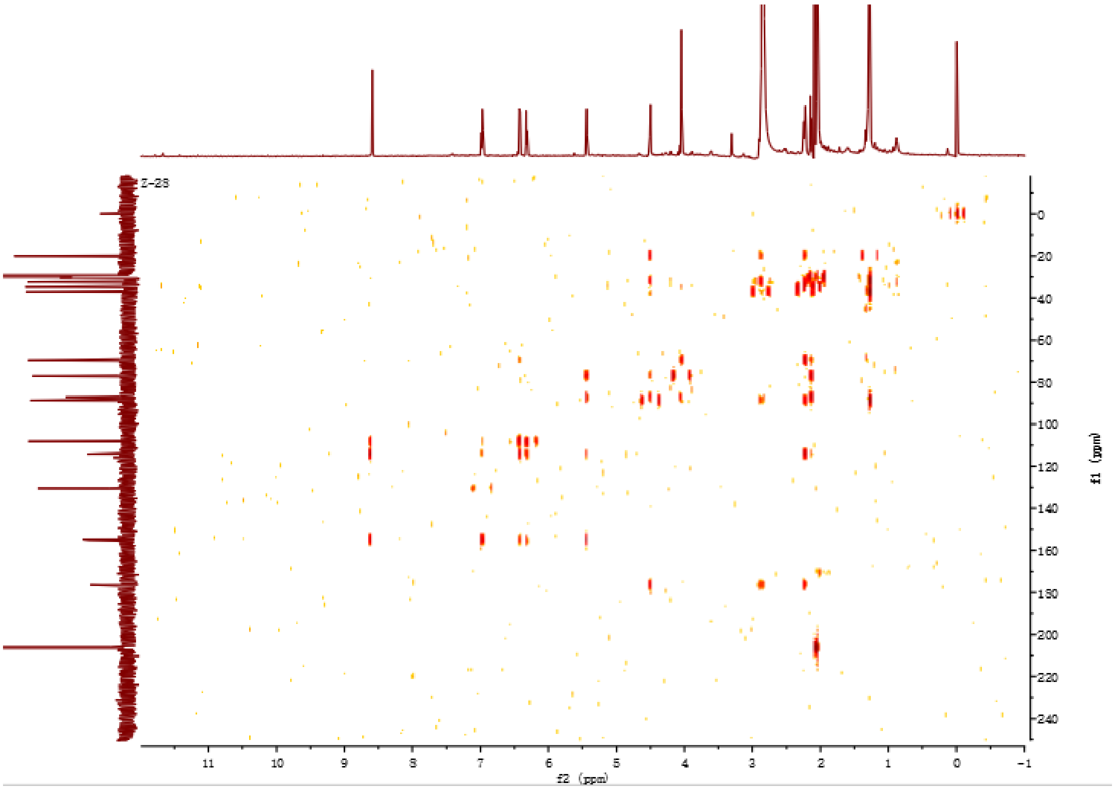

Figure S7. HMBC spectrum of Phaseolorin G (**1**)

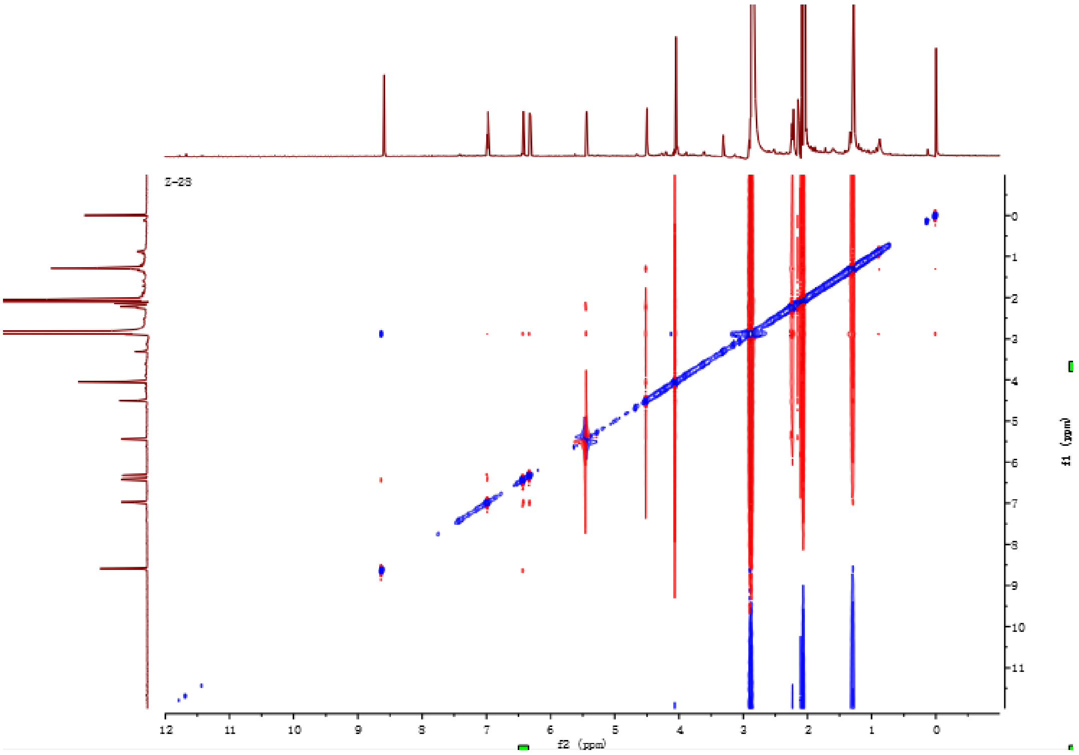

Figure S8. NOESY spectrum (600 MHz, acetone-d_6) of Phaseolorin G (**1**)

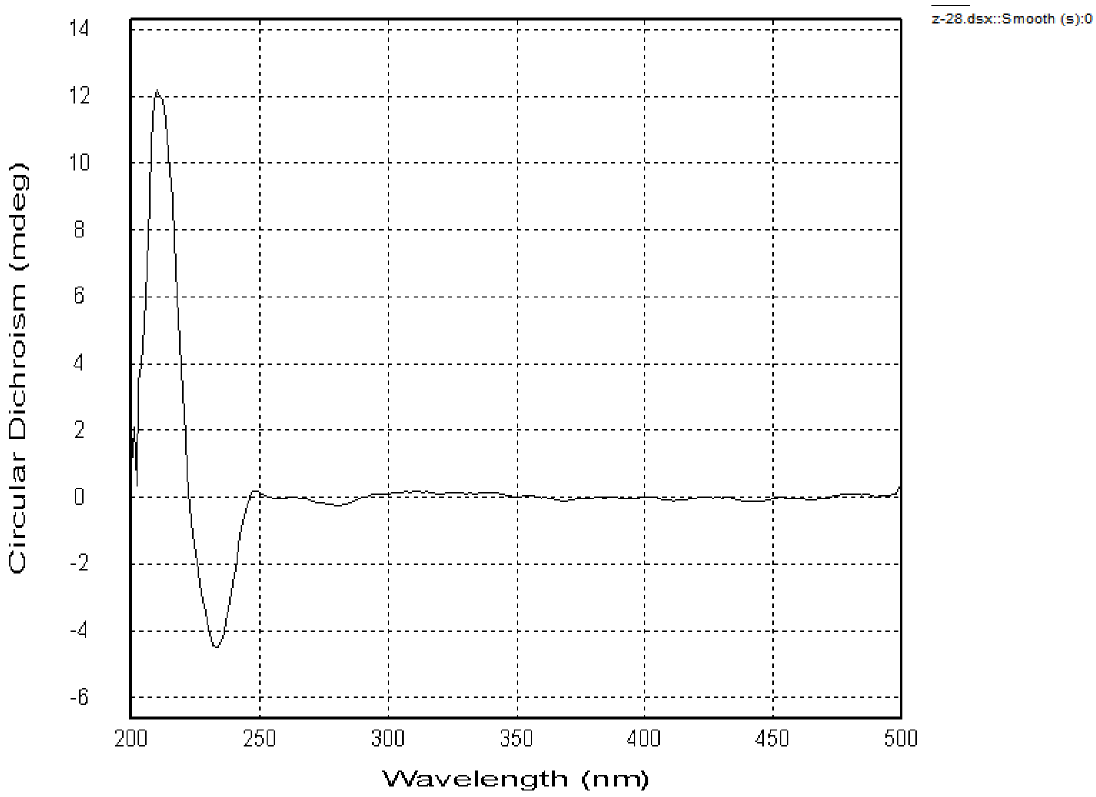

Figure S9. CD spectrum of Phaseolorin G (**1**)

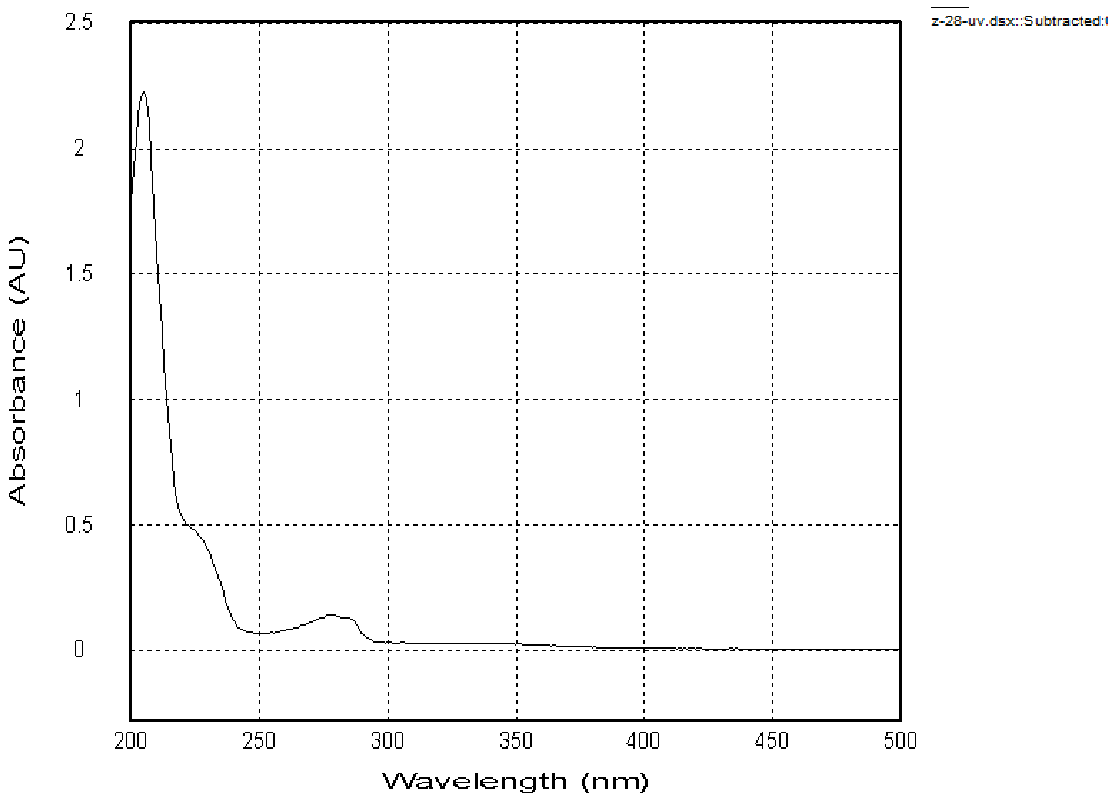

Figure S10. UV spectrum of Phaseolorin G (**1**)

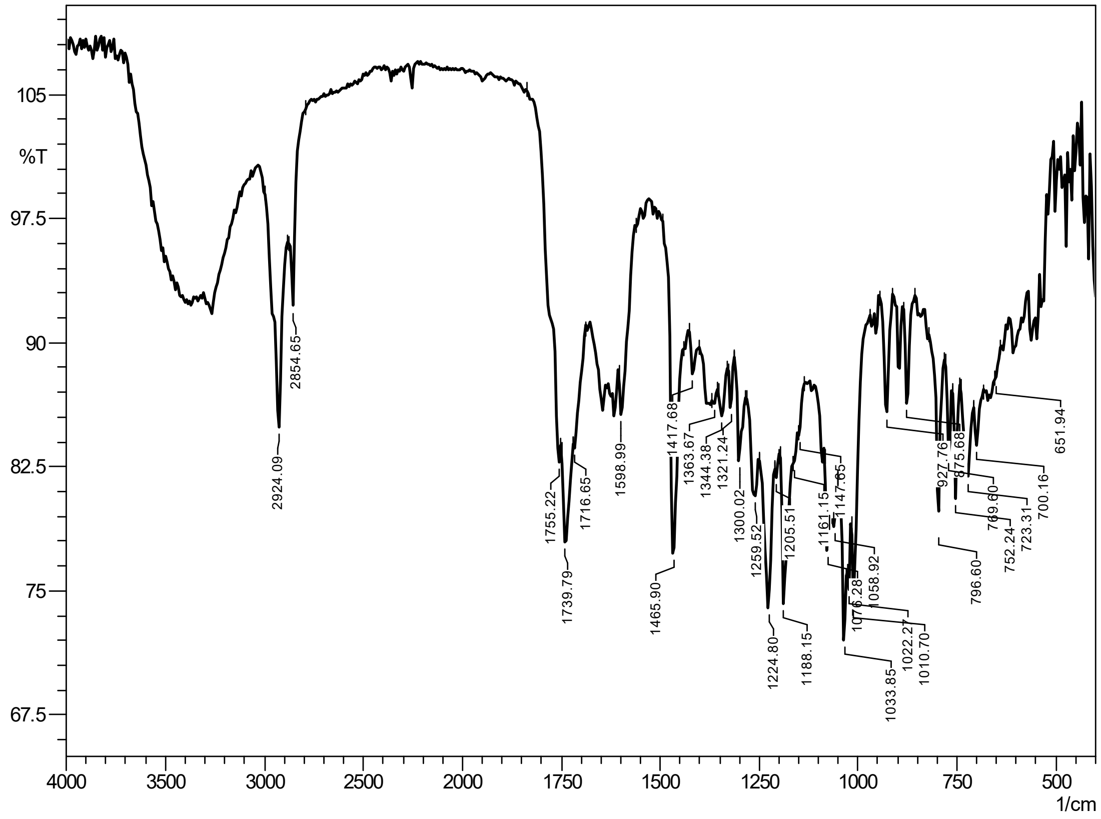

Figure S11. IR spectrum of Phaseolorin G (**1**)

Mass Spectrum SmartFormula Report

Analysis Info

Analysis Name	D:\Data\MS\data\201901\niuzheng_Z-8_pos_31_01_6042.d	Acquisition Date	1/4/2019 5:08:45 PM
Method	LC_Direct Infusion_pos_70-500mz.m	Operator	SCSIO
Sample Name	niuzheng_Z-8_pos	Instrument	maXis 255552.00029
Comment			

Acquisition Parameter

Source Type	ESI	Ion Polarity	Positive	Set Nebulizer	0.4 Bar
Focus	Active	Set Capillary	4500 V	Set Dry Heater	180 °C
Scan Begin	70 m/z	Set End Plate Offset	-500 V	Set Dry Gas	4.0 l/min
Scan End	1500 m/z	Set Charging Voltage	0 V	Set Divert Valve	Waste
		Set Corona	0 nA	Set APCI Heater	0 °C

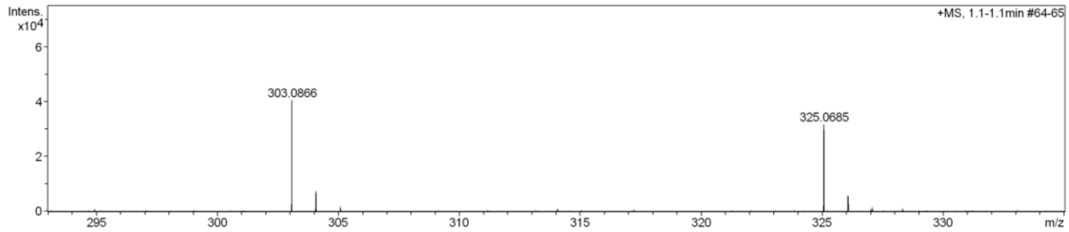

Meas. m/z	#	Ion Formula	Score	m/z	err [ppm]	err [mDa]	mSigma	rdb	e⁻ Conf	N-Rule
303.0866	1	C16H15O6	100.00	303.0863	0.8	0.3	2.0	9.5	even	ok
325.0685	1	C16H14NaO6	100.00	325.0683	0.7	0.2	2.7	9.5	even	ok
605.1635	1	C32H29O12	100.00	605.1654	-3.1	-1.9	23.5	18.5	even	ok
627.1476	1	C32H28NaO12	100.00	627.1473	-0.5	-0.3	29.1	18.5	even	ok

Figure S12. HRESIMS spectrum of Phaseolorin G (**1**)

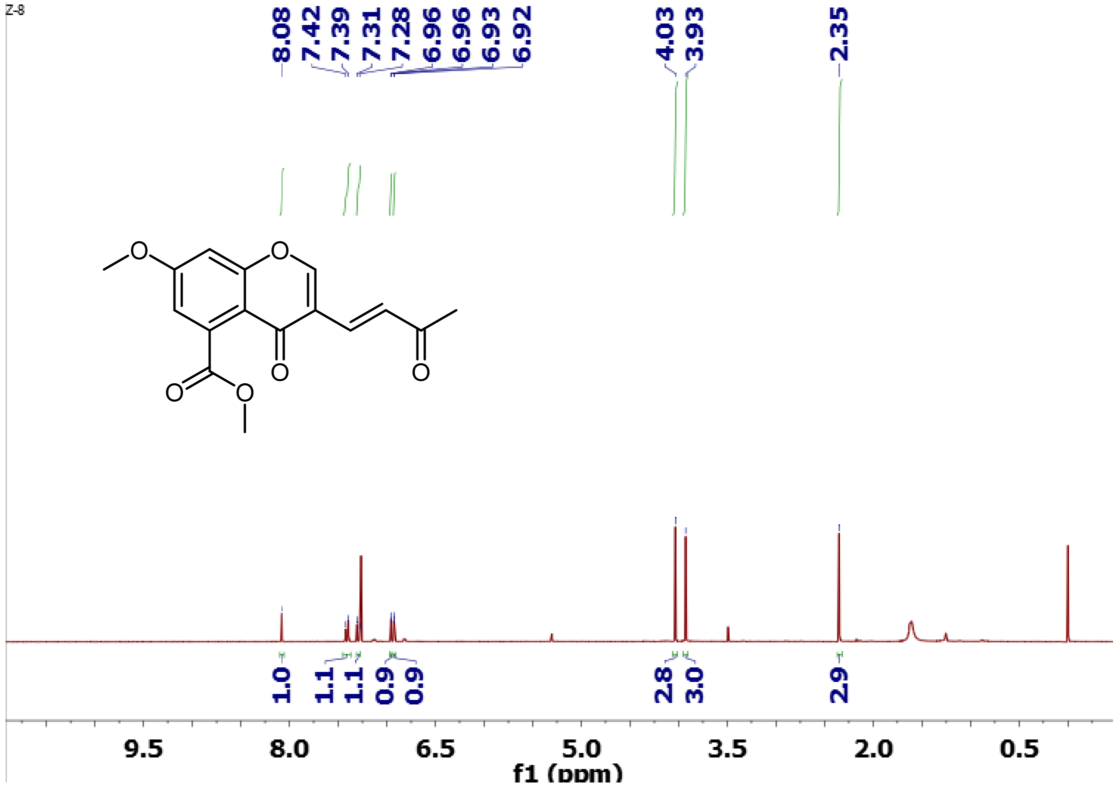

Figure S13. ^{1}H-NMR spectrum (600 MHz, $CHCl_3$) of Phaseolorin H (**2**)

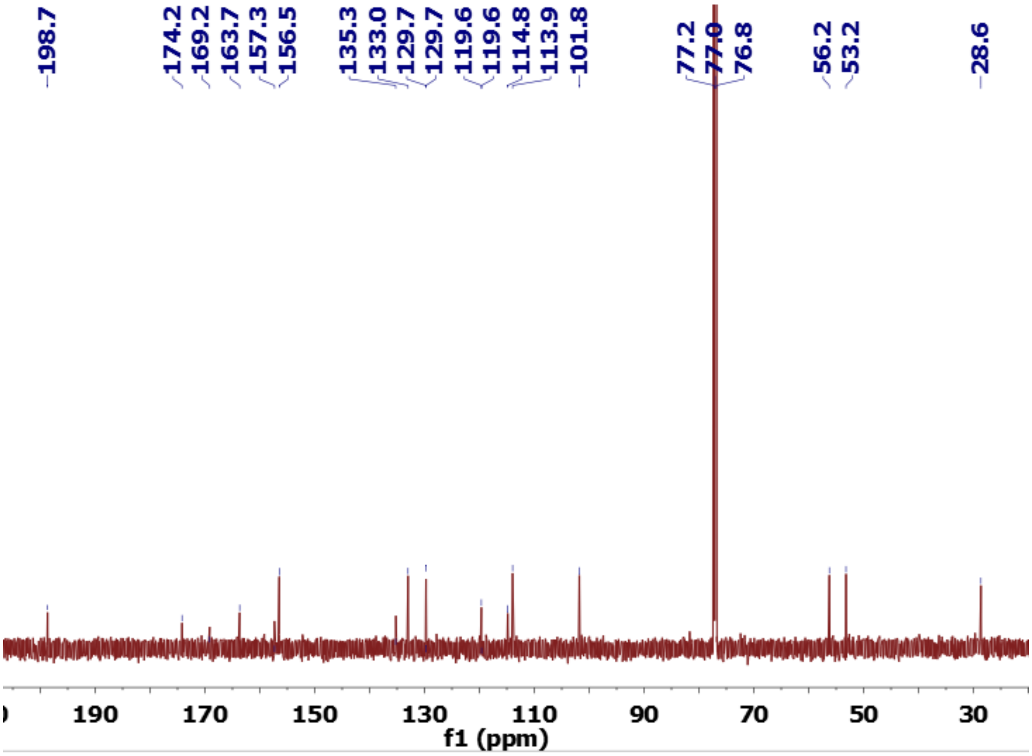

Figure S14. ^{13}C-NMR spectrum (150 MHz, $CHCl_3$) of Phaseolorin H (**2**)

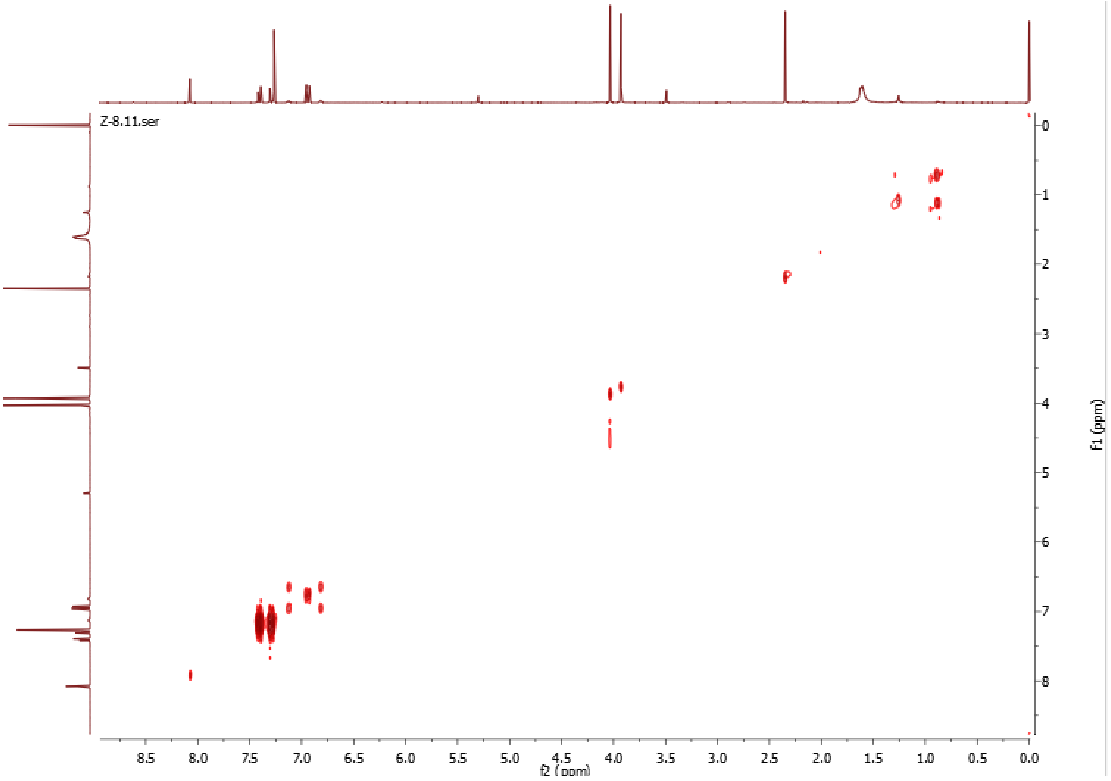

Figure S15. ^{1}H-^{1}H COSY spectrum (600 MHz, $CHCl_3$) of Phaseolorin H (**2**)

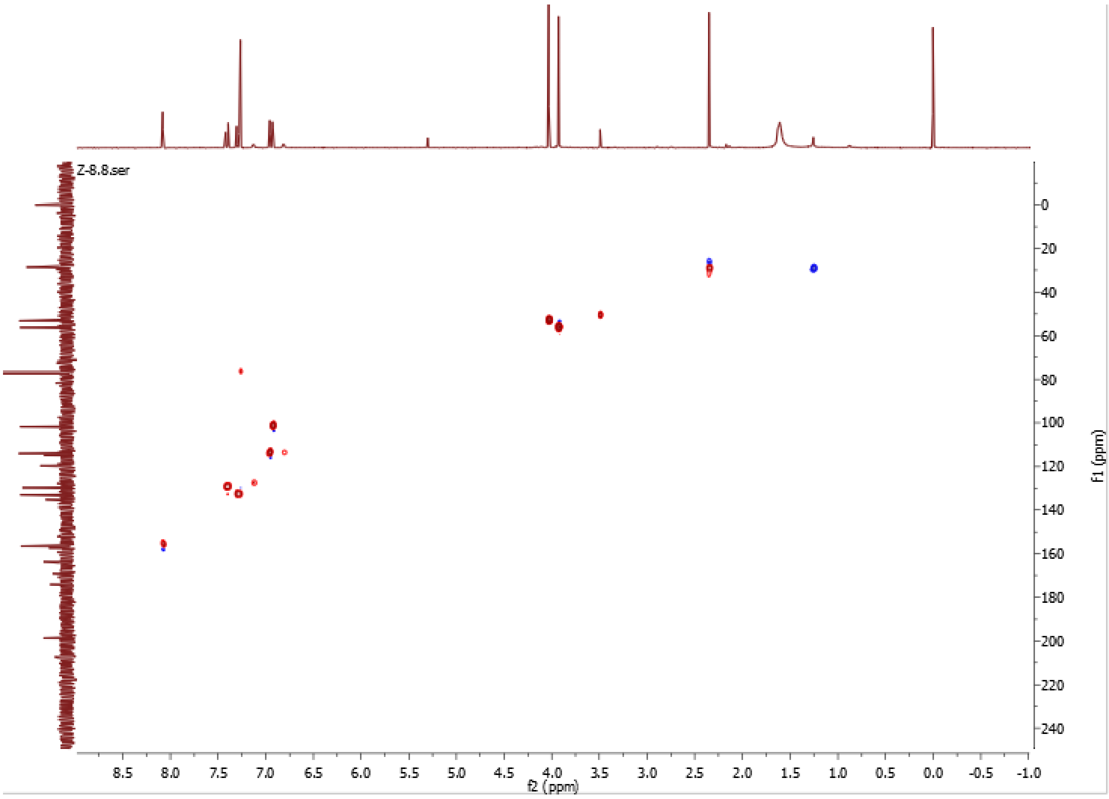

Figure S16. HSQC spectrum of Phaseolorin H (**2**)

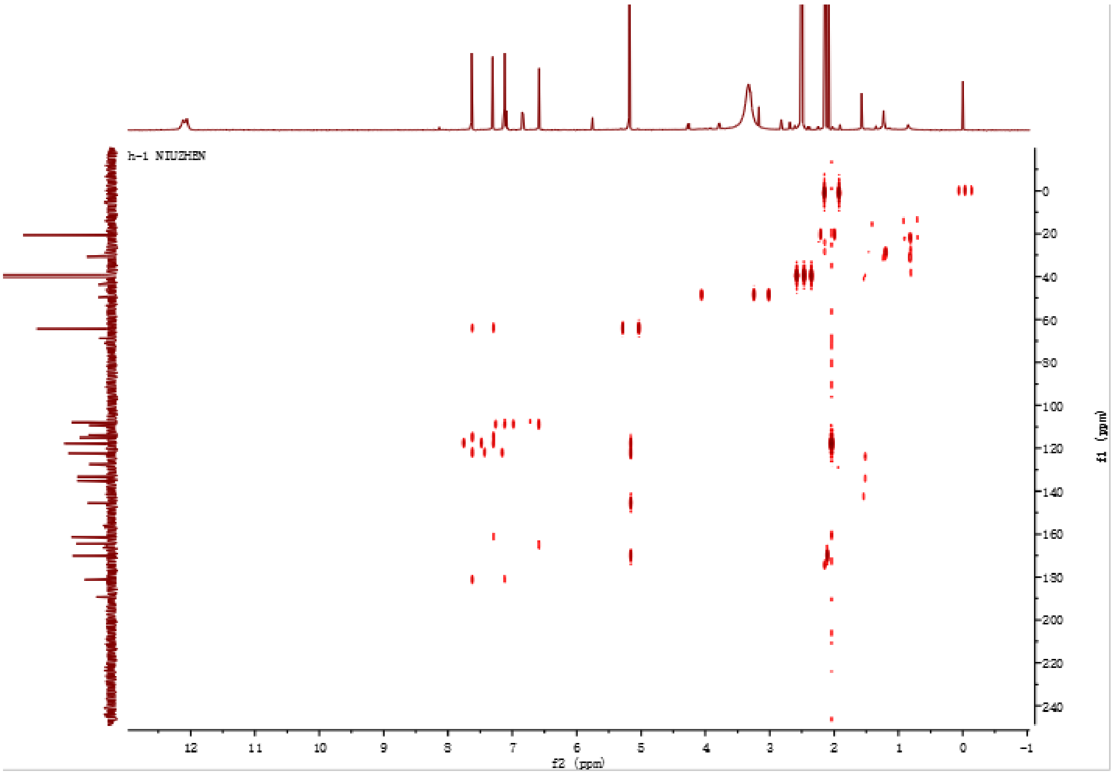

Figure S17. HMBC spectrum of Phaseolorin H (**2**)

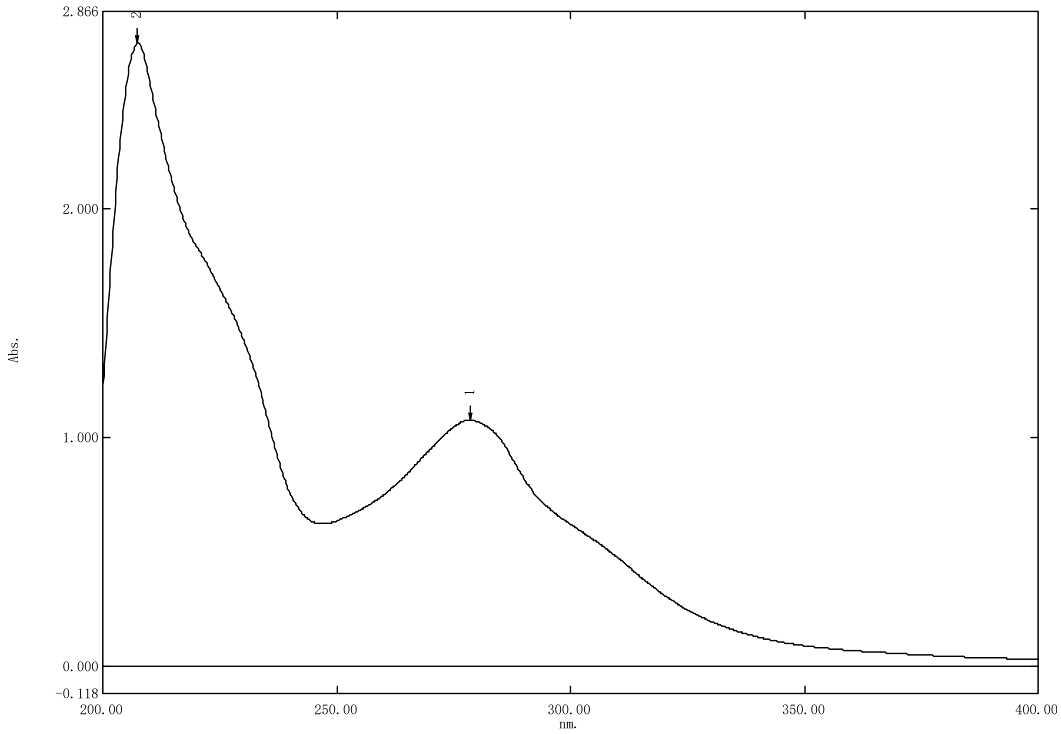

Figure S18. UV spectrum of Phaseolorin H (**2**)

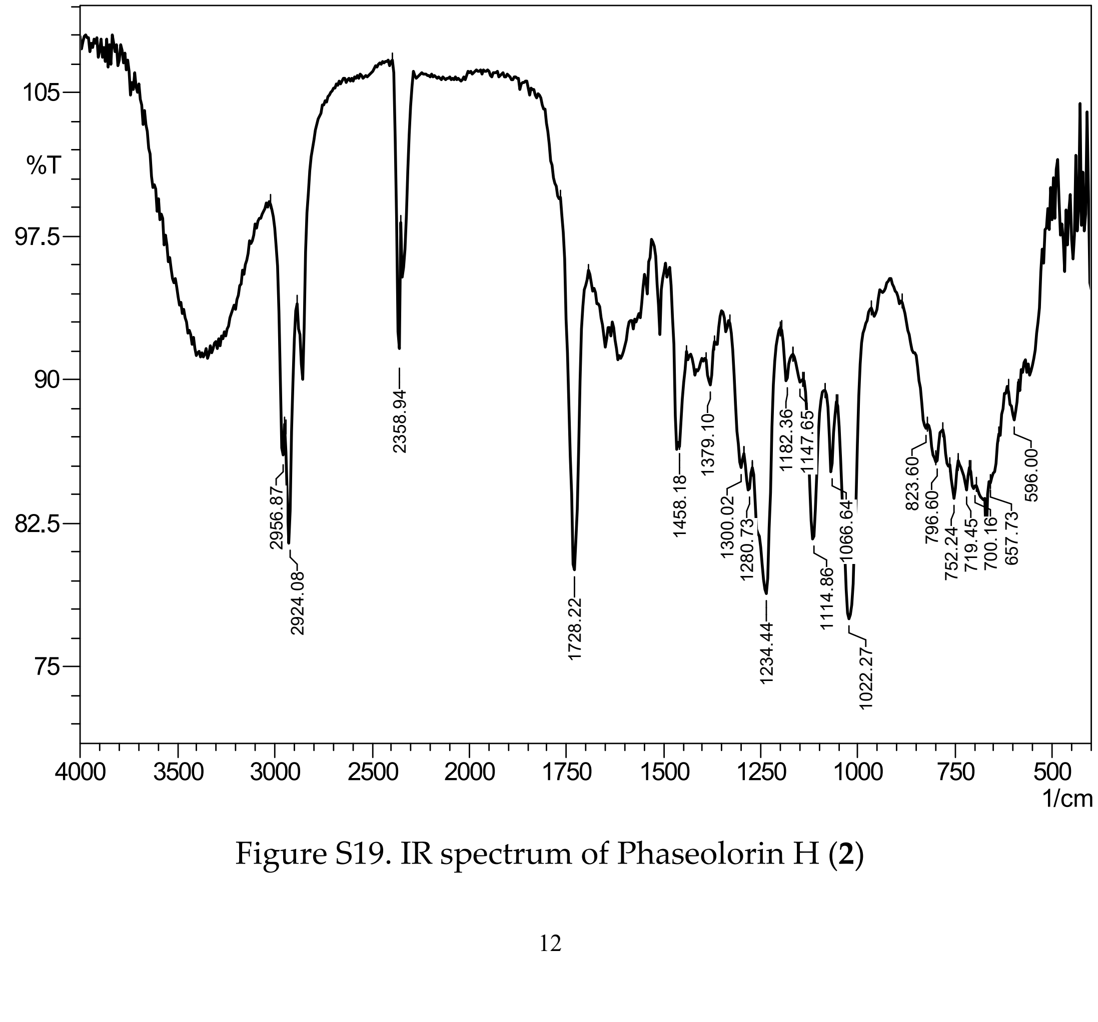

Figure S19. IR spectrum of Phaseolorin H (**2**)

Mass Spectrum SmartFormula Report

Analysis Info

Analysis Name	D:\Data\MS\data\201901\niuzheng_H-1_pos_32_01_6043.d	Acquisition Date	1/4/2019 5:12:12 PM
Method	LC_Direct Infusion_pos_70-500mz.m	Operator	SCSIO
Sample Name	niuzheng_H-1_pos	Instrument	maXis 255552.00029
Comment			

Acquisition Parameter

Source Type	ESI	Ion Polarity	Positive	Set Nebulizer	0.4 Bar
Focus	Active	Set Capillary	4500 V	Set Dry Heater	180 °C
Scan Begin	70 m/z	Set End Plate Offset	-500 V	Set Dry Gas	4.0 l/min
Scan End	1500 m/z	Set Charging Voltage	0 V	Set Divert Valve	Waste
		Set Corona	0 nA	Set APCI Heater	0 °C

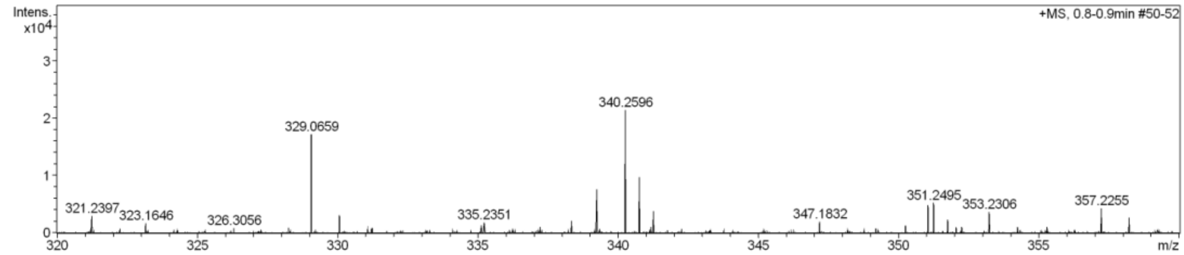

Meas. m/z	#	Ion Formula	Score	m/z	err [ppm]	err [mDa]	mSigma	rdb	e⁻ Conf	N-Rule
329.0659	1	C17H13O7	100.00	329.0656	1.0	0.3	5.6	11.5	even	ok
351.0479	1	C17H12NaO7	100.00	351.0475	1.1	0.4	12.2	11.5	even	ok

Figure S20. HRESIMS spectrum of Phaseolorin I (**3**)

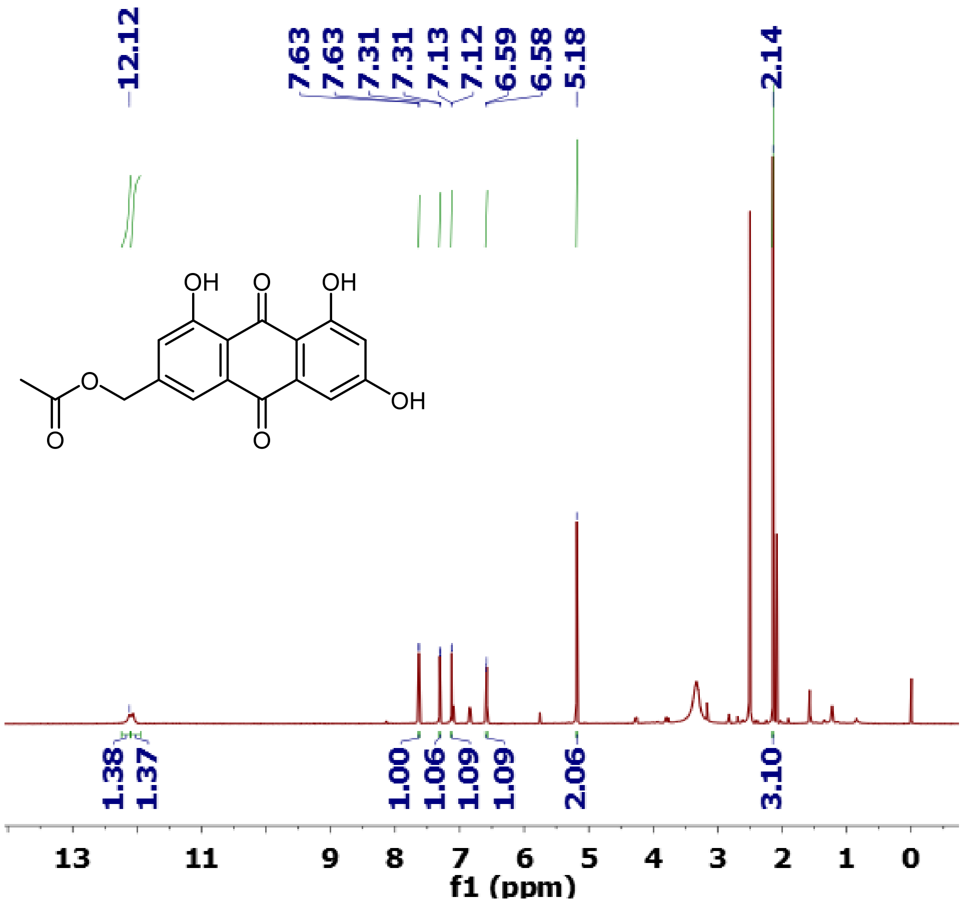

Figure S21. ^{1}H-NMR spectrum (600 MHz, DMSO-d_6) of Phaseolorin I (**3**)

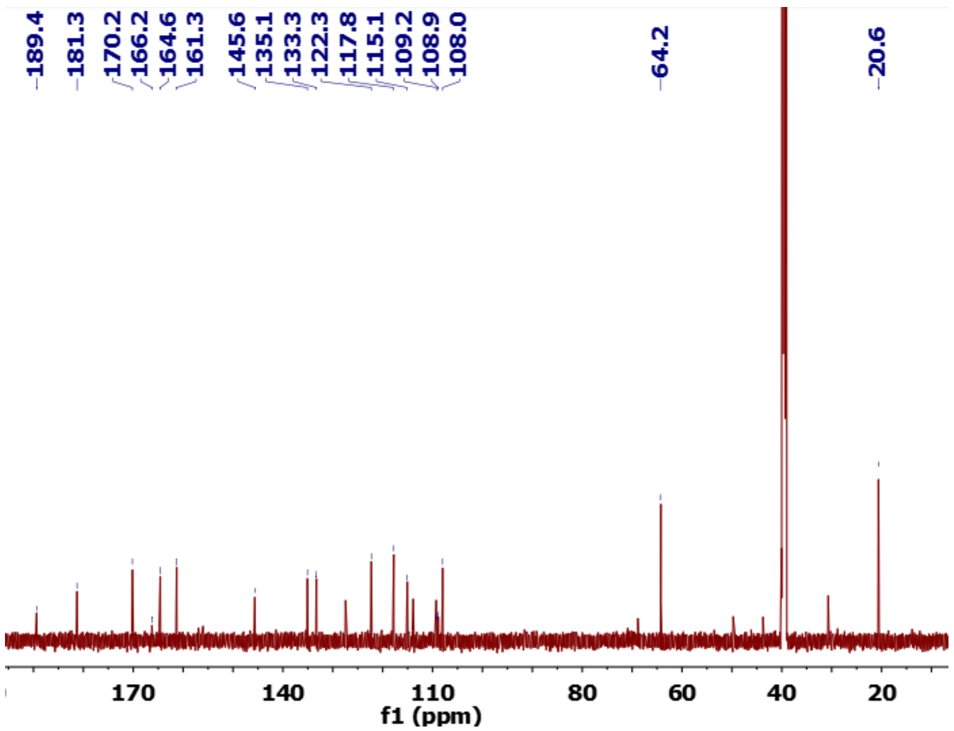

Figure S22. ^{13}C-NMR spectrum (150 MHz, DMSO-d_6) of Phaseolorin I (**3**)

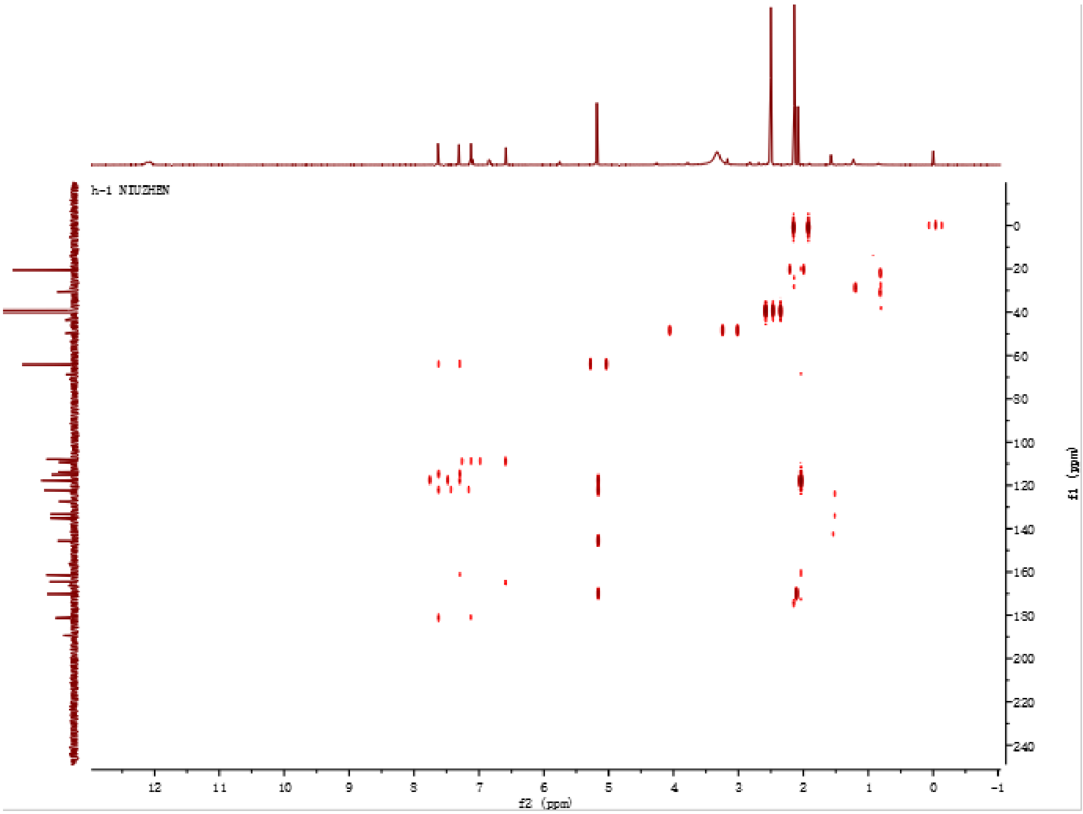

Figure S23. HMBC spectrum of Phaseolorin I (**3**)

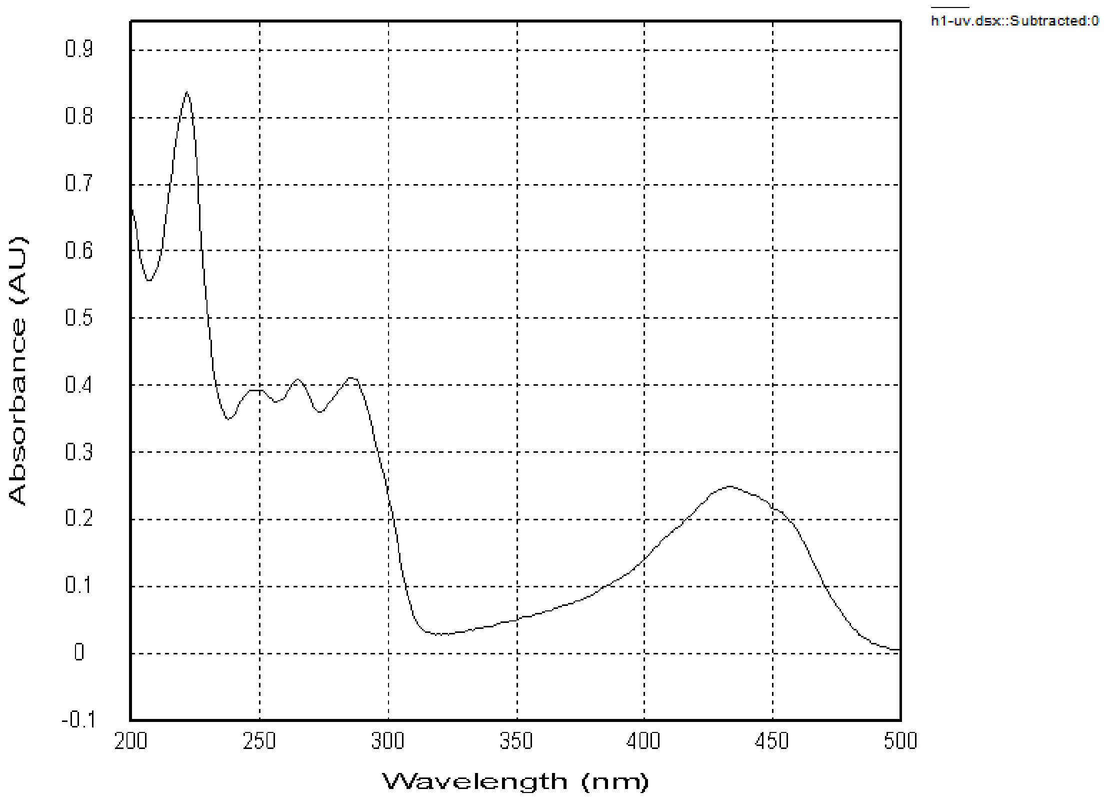

Figure S24. UV spectrum of Phaseolorin I (**3**)

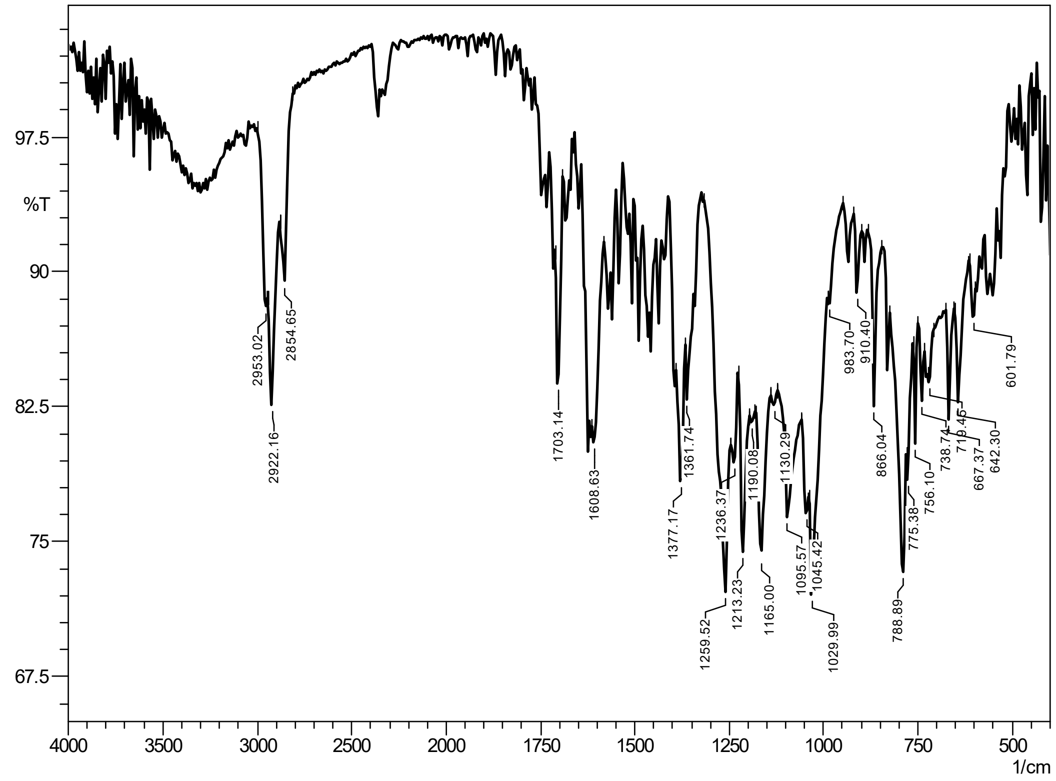

Figure S25. IR spectrum of Phaseolorin I (**3**)

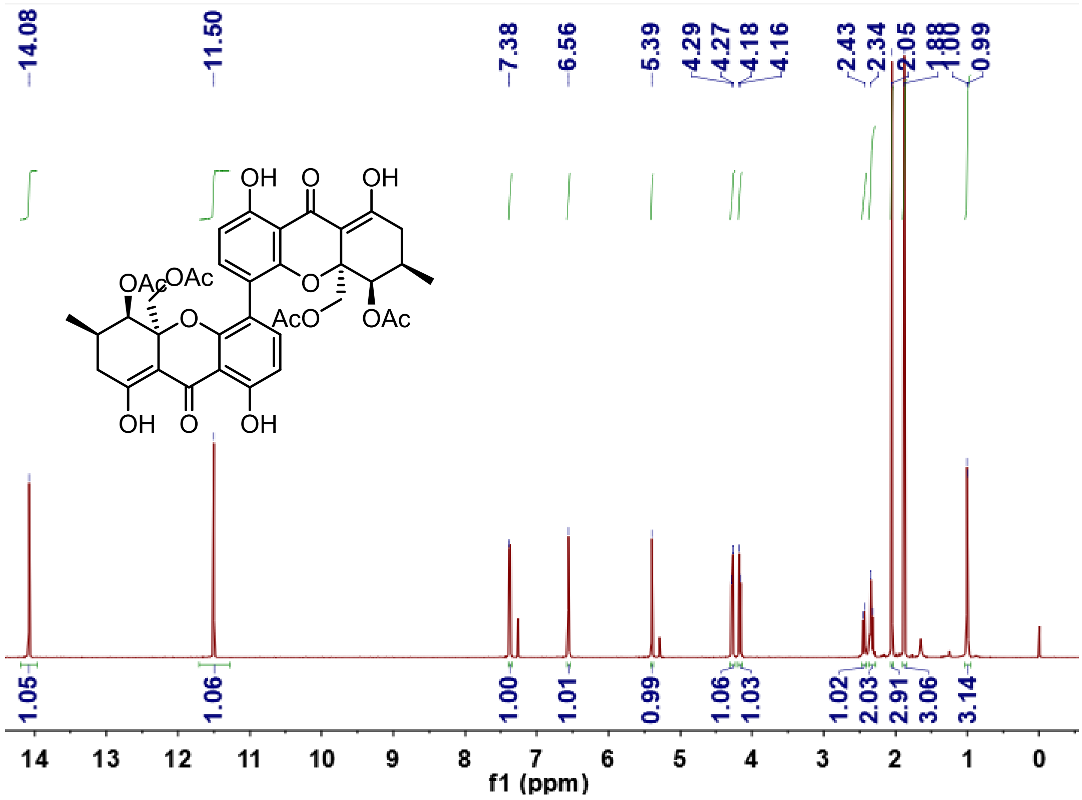

Figure S26. ^{1}H-NMR spectrum (600 MHz, $CHCl_3$) of Phomoxanthone A (**4**)

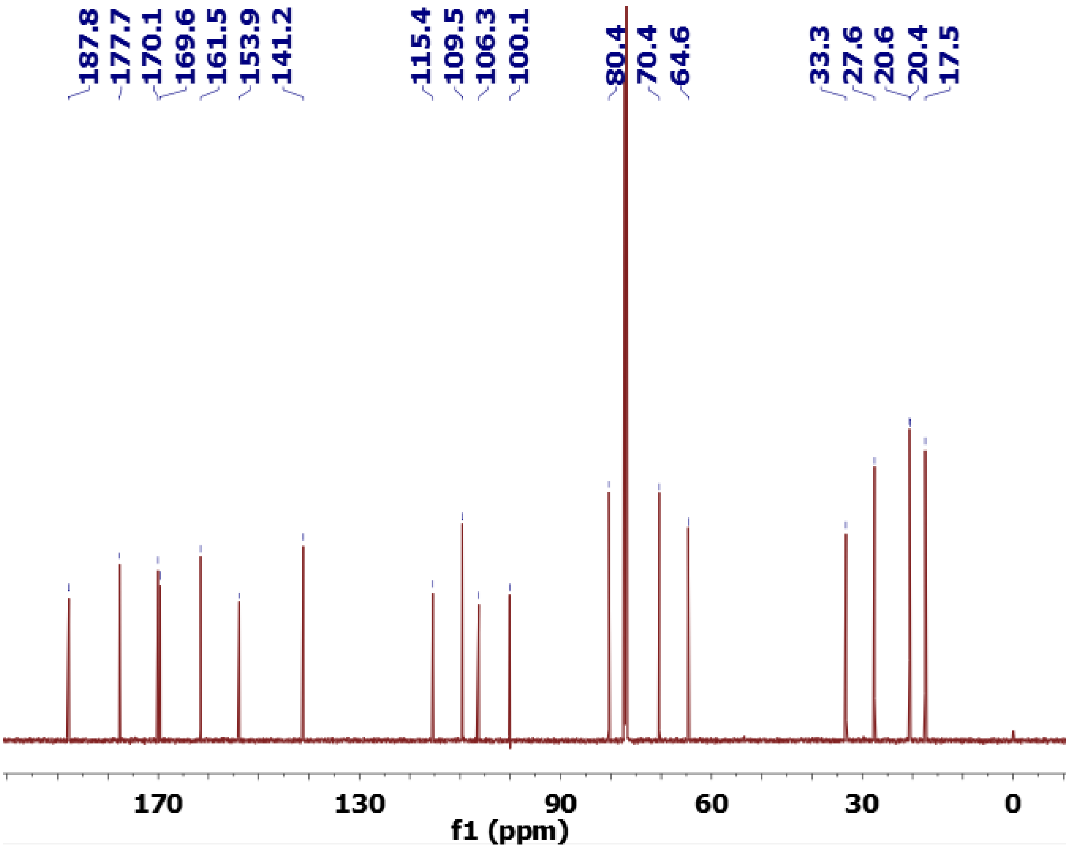

Figure S27. ^{13}C-NMR spectrum (150 MHz, $CHCl_3$) of Phomoxanthone A (**4**)

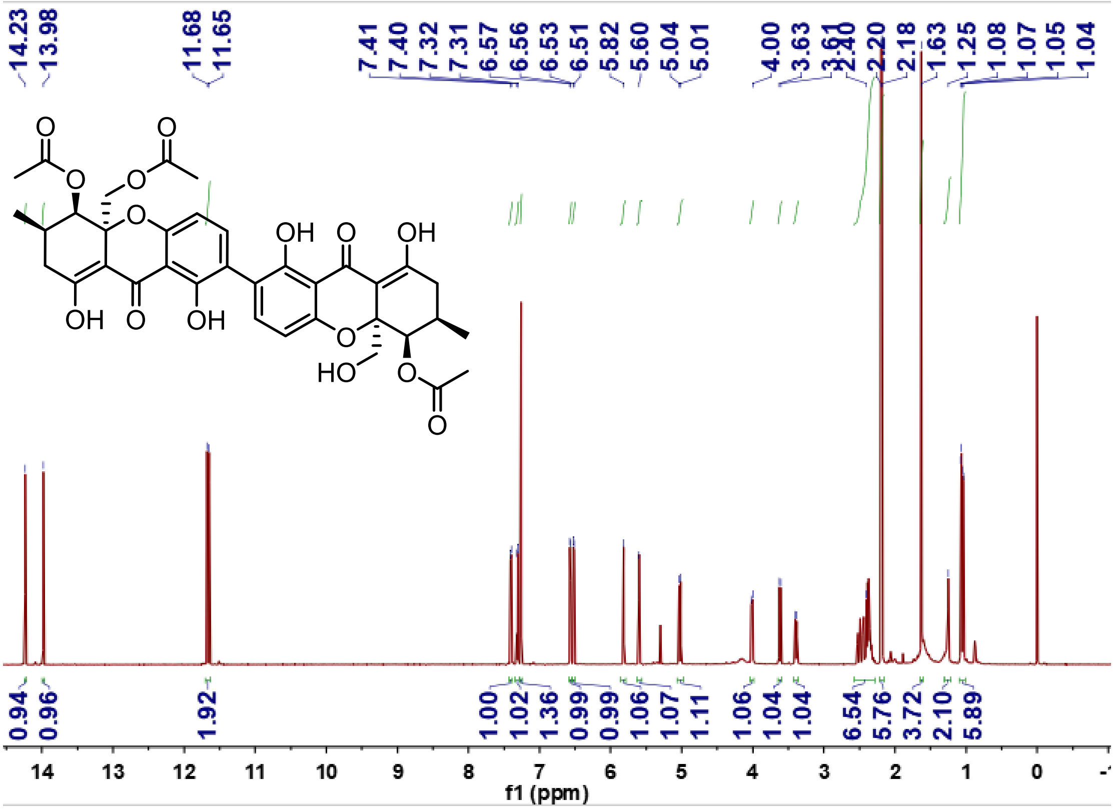

Figure S28. ^{1}H-NMR spectrum (600 MHz, $CHCl_3$) of Dicerandrol B (**5**)

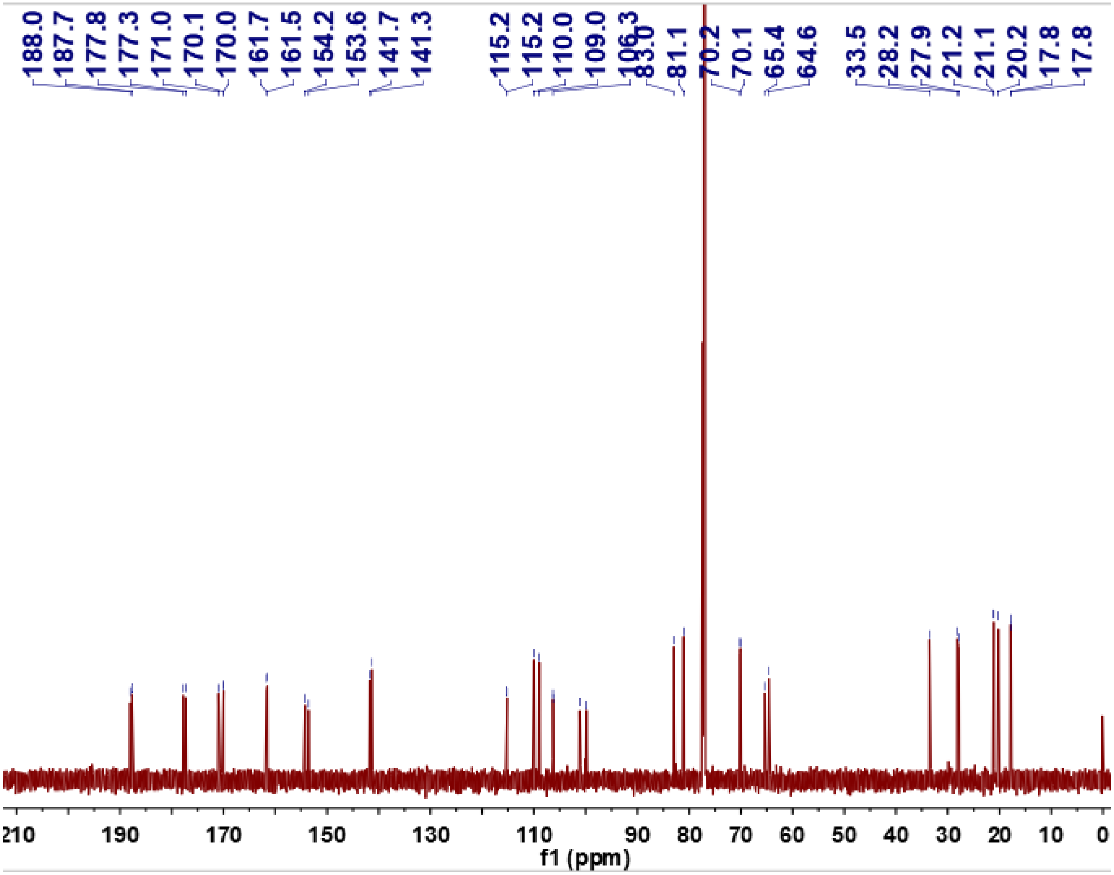

Figure S29. ^{13}C-NMR spectrum (150 MHz, $CHCl_3$) of Dicerandrol B (**5**)

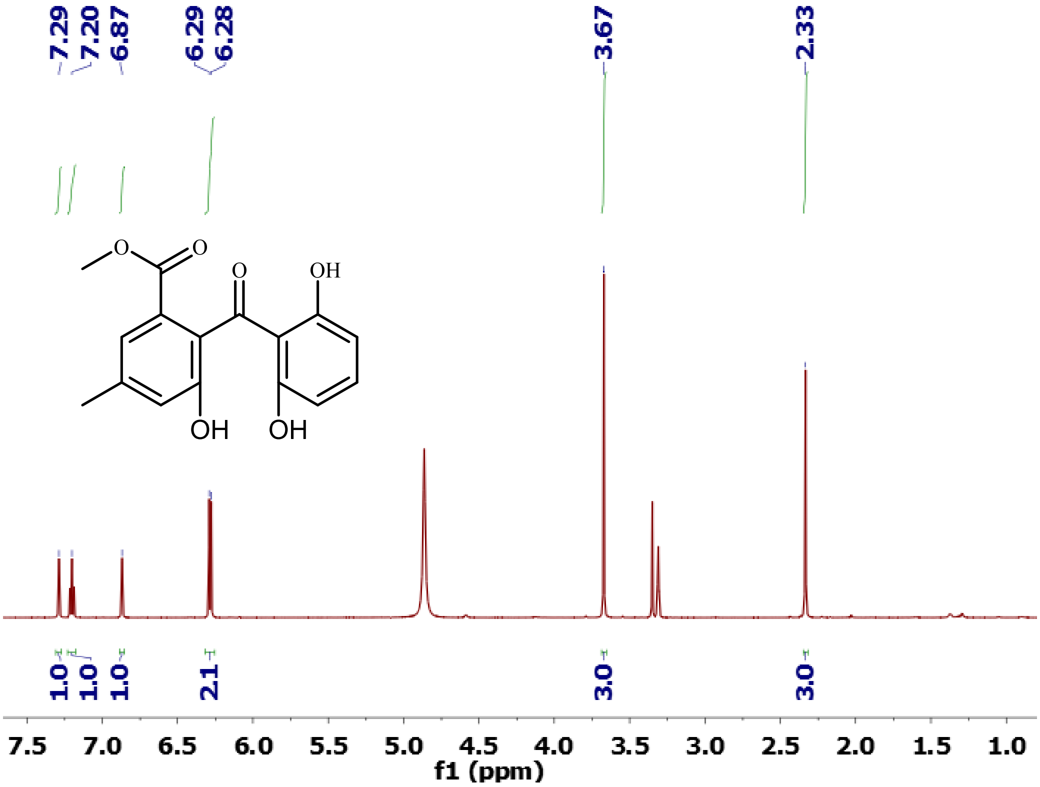

Figure S30. 1H-NMR spectrum (600 MHz, CD_3OD) of

2,2′,6′-trihydroxy-4-methyl-6-methoxy-acyl-diphenylmethanone (**6**)

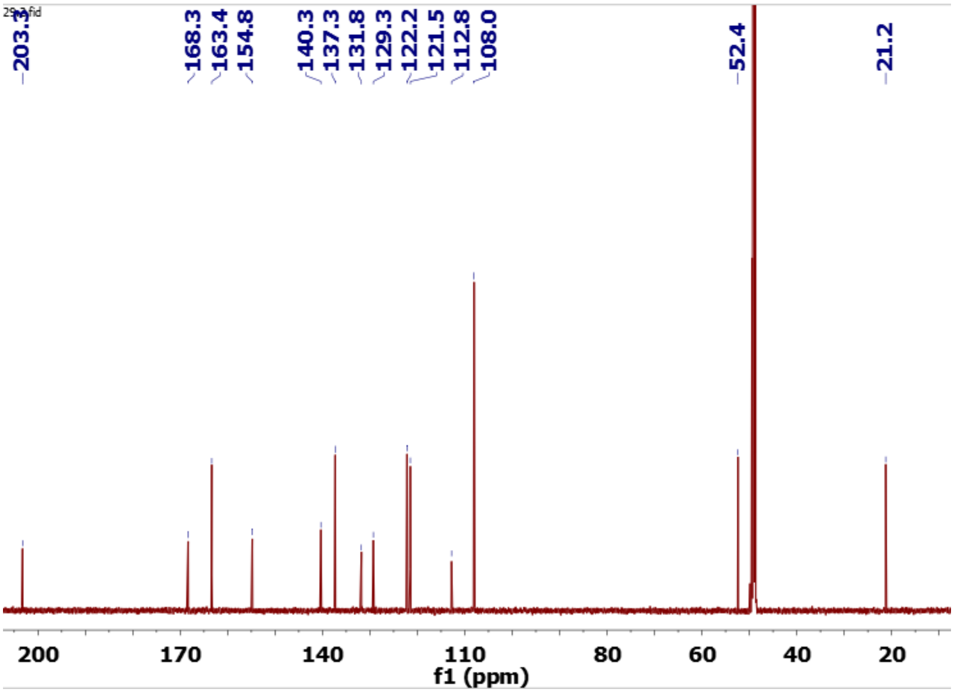

Figure S31. ^{13}C-NMR spectrum (150 MHz, CD_3OD) of

2,2′,6′-trihydroxy-4-methyl-6-methoxy-acyl-diphenylmethanone (**6**)

Table S1. Energy analysis for the Conformers of **1a** (**1**).

Compounds	CONFORMATION	E (Hartree)	E (Kcal/mol)	rel.E (Kcal/mol)	Boltzmann Dist (%)
**1a**	**1a**-1	-957.4240435	-600786.3638	0	76.40%
	**1a**-2	-957.42160243	-600784.832	1.531751521	5.75%
	**1a**-3	-957.42259761	-600785.4565	0.90727695	16.51%
	**1a**-4	-957.42023030	-600783.971	2.392771468	1.34%

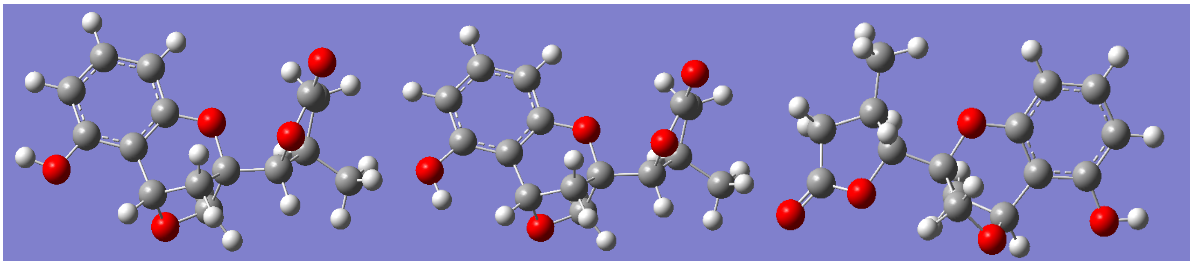

1a-1 **1a**-2 **1a**-3

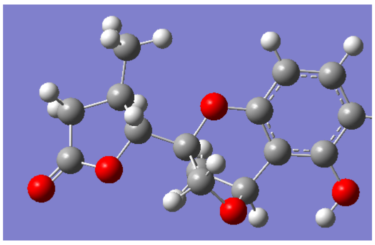

1a-4

Figure S32. B3LYP/6-31G(d,p) optimized low-energy conformers of **1a** (**1**)

Table S2. Energy analysis for the Conformers of **1b** (**1**).

Compounds	Conformation	E (Hartree)	E (Kcal/mol)	rel.E (Kcal/mol)	Boltzmann Dist (%)
**1b**	**1b**-1	-957.4240957	-600786.3966	0	76.65%
	**1b**-2	-957.42169738	-600784.8916	1.50497911	6.03%
	**1b**-3	-957.42261675	-600785.4685	0.928069259	15.99%
	**1b**-4	-957.42027066	-600783.9964	2.400248793	1.33%

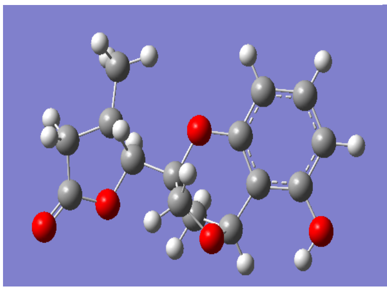

1b-1

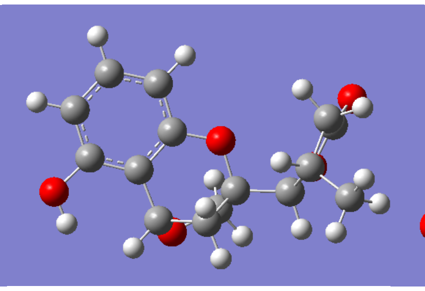

1b-2

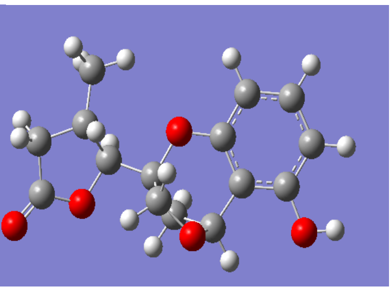

1b-3

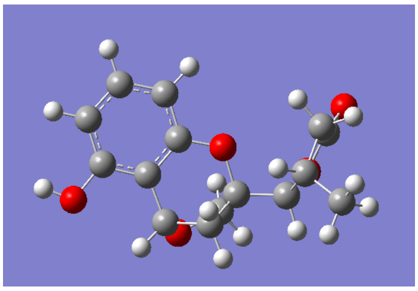

1b-4

Figure S33. B3LYP/6-31G(d,p) optimized low-energy conformers of **1b** (**1**)
